# Clinical Efficacy of Interventions Based on Professional Mechanical Plaque Removal in the Treatment of Dental Biofilm–Induced Gingivitis: A Systematic Review and Meta‐Analysis

**DOI:** 10.1111/jcpe.70083

**Published:** 2026-01-13

**Authors:** Roberto Farina, Anna Simonelli, Leonardo Trombelli, Ren Jie Jacob Chew, Yu‐Kang Tu, Philip M. Preshaw

**Affiliations:** ^1^ Research Centre for the Study of Periodontal and Peri‐Implant Diseases University of Ferrara Ferrara Italy; ^2^ Operative Unit of Dentistry Azienda Unità Sanitaria Locale (AUSL) Ferrara Italy; ^3^ Faculty of Dentistry National University of Singapore Singapore Singapore; ^4^ Institute of Health Data Analytics and Statistics, College of Public Health, and Health Data Research Center National Taiwan University Taipei Taiwan; ^5^ School of Dentistry University of Dundee Dundee UK; ^6^ School of Dental Sciences Newcastle University Newcastle upon Tyne UK

**Keywords:** dental plaque, dental prophylaxis, gingival diseases, gingivitis

## Abstract

**Aim:**

To evaluate the efficacy of professional mechanical plaque removal (PMPR) for treating naturally occurring dental biofilm–induced gingivitis (i) compared to no treatment or oral hygiene instructions (OHI) (FQ1), (ii) when performed through different modalities (FQ2) or (iii) when combined with professionally administered local adjuncts (FQ3).

**Materials and Methods:**

A structured literature search was conducted for randomised or non‐randomised controlled trials (RCTs and CTs) assessing gingival inflammation at patient level within 2–6 weeks after treatment in adults with gingivitis.

**Results:**

Heterogeneous evidence shows with low certainty that PMPR has no efficacy in patients continuing with ineffective self‐performed oral hygiene regimens but enhances OHI outcomes (FQ1; three RCTs, one CT). Split‐mouth RCTs consistently indicated with very low certainty that ultrasonic scaling (US) plus air polishing is as effective but less time consuming than US plus polishing with rubber cup and prophylaxis paste. Furthermore, diode laser shows no adjunctive benefit (FQ2; five RCTs). Although some professionally administered local adjuncts have shown positive outcomes in patients receiving PMPR, their broader clinical application is limited due to unresolved clinical issues and uncertain cost effectiveness (FQ3; two RCTs).

**Conclusions:**

OHI should be the first‐line treatment for dental biofilm–induced gingivitis. Combination of PMPR and OHI provides an adjunctive benefit over OHI alone. Air polishing may be combined with US to reduce the time for PMPR administration.

## 1 Introduction

Dental biofilm–induced gingivitis is acknowledged by the Classification of Periodontal Diseases and Conditions (Caton et al. 2018; Trombelli 2025) as a patient‐related disease entity characterised by an extent of gingival inflammation beyond a specific threshold (i.e., 10% bleeding on probing [BOP]) occurring on an intact or reduced periodontium in the absence of a history of periodontitis (Trombelli et al. 2018; Chapple et al. 2018). Reversion to a periodontally healthy condition (full‐mouth BOP score < 10%) currently represents the main treatment endpoint of gingivitis (Trombelli et al. 2018, Chapple et al. 2018). The rationale for gingivitis treatment comes from the impact that gingivitis has on patient perceptions of bleeding gums and from the oral health–related quality of life, especially when the disease is present in a generalised form (Krisdapong et al. 2012; Tomazoni et al. 2014; Kaewkamnerdpong et al. 2023; Al‐Bitar et al. 2024; Dikilitaş et al. 2024; Pupovac et al. 2024; Ripardo et al. 2025). However, and more importantly, gingivitis treatment is considered as the main strategy for the primary prevention of periodontitis (Chapple et al. 2015), gingivitis being regarded as the necessary precursor of periodontitis (Kinane and Attström 2005).

Along with the control of patient and local factors modulating the severity of the gingival inflammatory response to the dental plaque biofilm (Tatakis and Trombelli 2004; Trombelli et al. 2004, 2005, 2006; Trombelli and Farina 2013), mechanical plaque biofilm disruption and removal is the primary, essential component of gingivitis treatment, self‐administered by the patient and/or performed by an oral health professional (referred to as professional mechanical plaque removal [PMPR]). For the former, clear recommendations for oral hygiene instructions (OHIs) are currently available, consisting of behaviours including toothbrushing twice per day for at least 2 min with a fluoride toothpaste, interdental cleaning (mainly flossing in most gingivitis cases, due to the integrity of the interdental tissues, but otherwise interdental brushes, should space permit) once daily and, in some cases, implementing oral hygiene with agents for chemical plaque control (Needleman et al. 2015; Chapple et al. 2015). In this respect, two systematic reviews have recently shown that self‐performed mechanical oral hygiene, eventually implemented with chemical agents, may be effective in controlling gingivitis (Suvan et al. 2025; Figuero et al. 2025). Although it has been shown that OHI may be effective in controlling gingivitis irrespective of the additional use of PMPR (Hugoson et al. 2007), it can be speculated that in specific conditions OHI alone may be insufficient to restore gingival health, thus supporting the administration of PMPR in addition to OHI. In this respect, a single session of PMPR following OHI may successfully restore gingival health and improve self‐efficacy scores (Ying et al. 2025).

To date, there is a lack of clarity on when and how to administer PMPR to optimise the outcomes of gingivitis treatment. This consideration comes from the observation that a fraction of patients may not shift to a gingivally/periodontally healthy condition, with treatment success rates being partly associated with the baseline extent of gingivitis (Ying et al. 2025). Also, the scientific evidence on PMPR modalities in the treatment of gingivitis has never been comprehensively reviewed with a systematic approach to evaluate whether some PMPR interventions (when administered once) are superior to others in restoring periodontal health while potentially reducing the time required to undertake the procedure. Therefore, the present systematic review was performed to evaluate the efficacy of protocols based on PMPR for the treatment of naturally occurring dental biofilm–induced gingivitis, with emphasis on their rationale, modality (i.e., technique and/or technology) and implementation with the professional administration of adjuncts.

## 2 Materials and Methods

### 2.1 Focused Questions, Protocol Development and Registration

This present systematic review was performed to address the following focused questions (FQs):

− What is the clinical efficacy of PMPR in the treatment of gingivitis? (FQ1)
− Among the interventions based on PMPR, are some superior in terms of clinical outcomes, or do any allow for reduced treatment invasiveness while maintaining comparable clinical performance? (FQ2)
− Can the professional administration of adjunctive therapies enhance the clinical outcomes of PMPR‐based interventions? (FQ3)

An a priori protocol was developed, assessed and approved by the Scientific Committee of the 21st European Workshop on Periodontology. The systematic review was registered in PROSPERO (ID: 1017073) and follows the Preferred Reporting Items for Systematic reviews and Meta‐Analysis (PRISMA) (Liberati et al. 2009; Moher et al. 2009).

### 2.2 Study Selection Criteria

Study selection was based on the following inclusion criteria (PICOTS; Institute of Medicine [USA] 2011):

- Population: Adult populations consisting entirely or partly of subjects with naturally occurring dental biofilm–induced gingival inflammation on an intact or reduced periodontium in the absence of a history of periodontitis (e.g., traumatic gingival recessions), potentially including some periodontitis patients with maximum clinical attachment level (CAL) ≤ 3 mm or a maximum distance between the cemento‐enamel junction (CEJ) and the alveolar bone crest of ≤ 4 mm (if CAL was not assessed). A degree of flexibility was applied, whereby studies were considered eligible even when explicit reference to the participants' baseline diagnosis was not included among the inclusion criteria. In such cases, the study population comprised young adults, with no specific mention of periodontitis, yet the baseline inflammatory condition of the gingival tissues—although not explicitly listed as an inclusion criterion—was clearly described in the Results section of each study.
  Studies were excluded when dealing with experimentally induced gingivitis if they did not explicitly report either the age range or the baseline conditions of the periodontal support of the participants, if they were performed exclusively on periodontitis cohorts or if they were performed on cohorts consisting partly or entirely of periodontitis patients with CAL > 3 mm and/or distance CEJ–bone crest > 4 mm at any site; patients with drug‐induced gingival enlargement; patients with orthodontic appliances; patients with exposures to systemic diseases/conditions with documented impact on the outcomes of gingivitis treatment with PMPR; or patients with needs for special care to understand and/or perform home‐based oral hygiene.
- Intervention (for FQ1 and FQ2): Clinical procedures incorporating PMPR, where PMPR is defined as the professional, non‐surgical removal of dental plaque and calcified deposits, as performed using technologies with a prevalently mechanical, disrupting effect on the dental biofilm (e.g., sonic/ultrasonic/manual instrumentation; air‐abrasive devices) in single (full‐mouth) or multiple (partial‐mouth) sessions. The intervention may also incorporate risk‐factor control, either performed (e.g., elimination of plaque‐retentive local factors) or suggested (e.g., behavioural change) by the oral health professional; any measure to improve self‐performed plaque control (e.g., OHI, provision of devices) either prior to PMPR or within the same appointment; (for FQ3): Clinical procedures incorporating PMPR and adjunctive therapies administered locally by an oral health professional in the attempt to enhance PMPR effects through debris removal (e.g., water irrigation), an alteration of biofilm structure (e.g., desiccants) or an antimicrobial effect (e.g., chlorhexidine, photodynamic therapy).
- Comparison (for FQ1): No treatment, that is, continuation of routine oral hygiene procedures (if PMPR was administered without OHI in the Intervention) or OHI alone (if PMPR was administered with OHI in the Intervention); (for FQ2): Any of the aforementioned Interventions; (for FQ3): Same as intervention, but without professional administration of adjunct/s.
- Outcome measures: Although primary interest was given to the proportion of patients reverting from gingivitis (as defined by Chapple et al. 2018; Trombelli et al. 2018) to periodontal health (Chapple et al. 2018, Trombelli et al. 2018), any clinical measure of gingival inflammation expressed at the patient level (e.g., BOP score, gingival index) as either a post‐treatment change or a value at the follow‐up visit was considered among the primary outcomes. For studies reporting data on at least one primary outcome measure, supragingival plaque scores and treatment invasiveness were also considered as secondary outcome measures. For the latter, a broad definition was used, including intra‐ and post‐operative morbidity, complications, incidence of adverse events/harms either local or systemic (in case of adjuncts), chair time for treatment administration, costs related to the procedure and patient‐reported outcomes (Farina et al. 2023). Studies that assessed outcomes only after repeated administrations of the intervention at regular intervals were included but used only for data following the first administration; if this was not possible, they were excluded.
- Timing: Studies including the assessment of a primary outcome between 2 and 6 weeks following the completion of the administration of the Intervention.
- Study design: Prospective randomised controlled trials (RCTs) or controlled trials (CTs) with a split‐mouth or parallel‐arm design. Only studies with a per protocol population of at least five subjects per investigated treatment were considered.

### 2.3 Literature Search

#### 2.3.1 Electronic and Hand Searches

According to the strategy reported in Appendix S1, Medline, Elsevier Scopus and Embase databases were searched for relevant literature from inception up until 2 May 2025. A separate search of the Cochrane Oral Health Group Specialty Trials' Register was conducted, and the reference lists of relevant systematic reviews were screened for the presence of eligible studies. Grey literature was retrieved through the Proquest database. The *Journal of Clinical Periodontology*, the *Journal of Periodontology*, the *Journal of Periodontal Research* and the *Journal of Dental Research—Clinical and Translational Research* were hand‐searched. The reference lists of the selected publications were screened for the presence of eligible studies. No language restrictions were applied. Titles and abstracts from the electronic searches were managed using Zotero (v7.0.7; Corporation for Digital Scholarships).

#### 2.3.2 Screening Methods

Duplicates were removed using the Rayyan software (Cambridge, MA). Two authors (A.S. and J.C.) performed the primary search by independently screening the titles and abstracts. The same reviewers selected full manuscripts for studies meeting the inclusion criteria. After identifying the studies to be included, the authors resolved disagreements by discussion. If consensus was not reached, disagreements were resolved by discussion with a third author (P.M.P.).

#### 2.3.3 Data Extraction: Intervention Characterisation

Two reviewers (A.S. and J.C.) extracted data in duplicate and resolved disagreements by discussion. For each included study, data were retrieved and recorded using specially designed forms.

#### 2.3.4 Assessment of Reporting Quality and Risk of Bias in Individual Studies

Adherence to reporting standards was recorded for each study.

For the included RCTs, a methodological quality assessment was performed according to the revised Cochrane Risk of Bias tool (ROB 2.0) for randomised trials (Sterne et al. 2019). Five main domains for risk of bias were assessed: randomisation process, deviations from the intended interventions, missing outcomes data, measurement of the outcomes and selection of the reported results. A risk of bias judgement (among ‘low risk of bias’, ‘some concerns’, or ‘high risk of bias’) was assigned to each domain (depending on the descriptions given for each field) or to the entire study.

For the included CTs, a methodological quality assessment was performed according to the Cochrane risk of bias tool (ROBINS‐I) in non‐randomised studies of intervention (Sterne et al. 2016). The risk of bias was assessed for seven main domains: bias due to confounding, selection bias, classification of interventions, deviations from the intended interventions, missing outcome data, measurement of outcomes and selection of reported results. A risk of bias judgement (among ‘low’, ‘moderate’ or ‘serious’ risk of bias) was assigned for each domain and for the overall risk of bias of each included CT.

##### 2.3.4.1 Certainty Assessments

The quality of evidence and the strength of recommendations were evaluated by using the Grading of Recommendations Assessment, Development and Evaluation (GRADE) (Guyatt et al. 2013; Meader et al. 2014). For each meta‐analysis, the certainty of the evidence was evaluated based on the inconsistency of results, indirectness of the evidence, imprecision and other considerations, which include publication bias, the effect size (ES), plausible confounding factors and the presence of dose–response effects. Inconsistency refers to unexplained heterogeneity in the results, potentially due to the differences in population, interventions, controls and outcomes among the contributing studies. The indirectness of evidence refers to the differences in population, intervention, comparison and outcomes of the contributing studies, compared to the review question. Imprecision refers to reduced certainty about the effect estimates due to wide confidence intervals or small sample sizes.

Both assessments were performed independently by two reviewers (R.J.J.C. and P.M.P.). In case of any discrepancies between the two reviewers, further discussion was conducted to reach a consensus. If a disagreement persisted, the final decision was made by a third reviewer (R.F.).

#### 2.3.5 Statistical Analysis

Data from the included studies were used to characterise treatment effects at the 2–6‐week observation interval. Each study contributed to this interval once per outcome parameter. Therefore, for studies reporting multiple observations within this interval, data from the 3‐week observation were preferentially extracted. If a study did not report data at 3 weeks, preference was given to other data in the following order: 4, 2, 5 and then 6 weeks. When available, data on the long‐term effects of gingivitis treatment (> 6 weeks) were also extracted and reported separately.

For pairwise meta‐analyses, we intended to use the common‐effect model when between‐study heterogeneity was small; otherwise, the random‐effects model with the Simonian–Laird method for estimating the between‐study variance would be used. However, if the number of included studies was < 5, we used the common‐effect model because the number of included studies was too small to obtain a robust estimate of the heterogeneity variance for the random‐effects model (Bender et al. 2018). The effect size measure was the weighted mean difference when the included studies reported the same indices for gingival bleeding or dental plaque. If different indices were reported, the standardised mean difference was used as the effect size measure.

For continuous outcomes, we extracted the change in the bleeding or plaque indices from baseline, the standard deviation and the number of patients in each treatment group from the included studies. If a study only reported the mean values at baseline and follow‐up, we calculated the mean change scores for each treatment group by subtracting the mean of the follow‐up from the mean of the baseline. The standard deviation was derived by assuming a correlation of 0.5 between the baseline and follow‐up values. When a study made within‐person comparisons (e.g., in the split‐mouth design), we assumed a within‐person correlation of 0.25 for calculating the standard deviation of the between‐group mean differences (Lesaffre et al. 2009).

If a study did not report the standard deviation, we used the following strategy to impute the missing data. When a *p*‐value for a comparison was reported, we used the *p*‐value to obtain the corresponding *t*‐value by using the cumulative *t*‐distribution function in the free R statistical software. Then, the formula for computing the *t*‐statistic was used to derive the standard deviation (SD):

$$t=\frac{\text{mean}}{\sqrt{\frac{\mathrm{SD}}{n}}},$$

where *n* is the sample size. If the significance level alone was reported, such as *p* < 0.05 or *p* < 0.001, we used *p* = 0.05 or *p* = 0.001 in the formula. If a comparison was statistically non‐significant but the *p*‐value was not available, we used *p* = 0.5 to derive the SD by assuming that the *p*‐value followed a uniform distribution over the interval of [0, 1] under the null hypothesis.

For studies that used sites as the unit of analysis, we made an adjustment to the calculation of the standard error in each treatment group. The effective sample size for a study in which each patient contributed more than one tooth or site ranges from the number of patients (the minimum) to the number of teeth/sites (the maximum). We, therefore, used the number of patients in the calculation of the standard error (SE) to obtain a conservative estimate:

$$\mathrm{SE}=\frac{\mathrm{SD}}{\sqrt{\text{number of patients}}}$$

This effectively assigns a smaller weight to the study that reported site‐level analyses (Albonni et al. 2021).

All statistical analyses were undertaken using the ‘meta’ package for the free statistical software R (version 4.5.1, R Foundation for Statistical Computing, Vienna, Austria) (Balduzzi et al. 2019).

## 3 Results

### 3.1 Search Results and Description of the Included Studies

**FIGURE 1:**
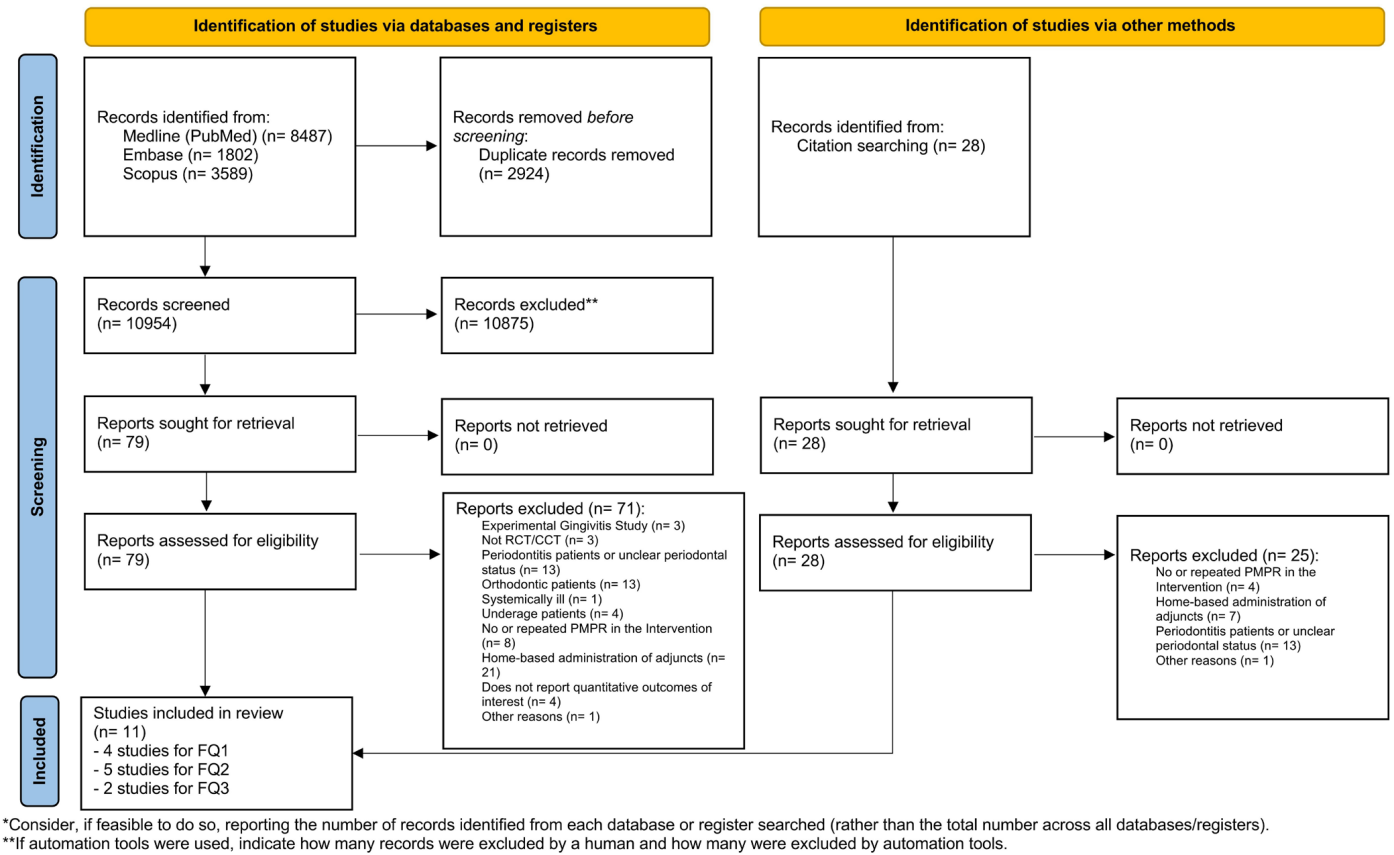
PRISMA flow diagram of study screening and selection.

The screening and selection process of the study (Figure 1) resulted in the inclusion of 11 studies. Among these, four, five and two studies were pertinent to FQ1 (Table 1a), FQ2 (Table 2a) and FQ3 (Table 3a), respectively.

**TABLE 1a:** FQ1: Characteristics of RCTs and CTs evaluating the efficacy of PMPR alone with reference to no treatment (i.e., continuation of routine oral hygiene) or PMPR + OHI with reference to OHI alone.

Study	Study design and financial support	Per protocol population (referred to the longest follow‐up visit reported in this Table)					Treatment					Study outcomes and analysis		Follow‐up (in addition to the 2–6 week interval)	Primary outcomes (gingival inflammation scores)	Secondary outcomes
		Participants	*n*	Age (years)	Sex	Smoking	PMPR modality	PMPR extension below the gingival margin	Operator performing PMPR	Type of OHI	Timing of OHI	Sites for outcome assessment	Statistical unit			
Tan and Saxton (1978)	Parallel‐arm RCT (supported by Industry)	Volunteers with gingivitis selected among army recruits	25	NR	NR	NR	Scaling with ultrasonic‐magnetostrictive instruments + fine curettes + Orban file + rubber cup + bristle brush and pumice	NR	Certified dental hygienists	Group discussion (30 min), verbal + audiovisual personal instruction (average 10 min) + provision of oral hygiene devices	After initial examination	Full‐mouth^a^	Patient	3 months	GBS	Plaque score^b^
			22	NR	NR	NR	No PMPR	—	—	Group discussion (30 min), verbal + audiovisual personal instruction (average 10 min) + provision of oral hygiene devices	After initial examination					
			27	NR	NR	NR	Scaling with ultrasonic‐magnetostrictive instruments + fine curettes + Orban file+ rubber cup + bristle brush and pumice	NR	Certified dental hygienists	—	—					
			24	NR	NR	NR	No PMPR	—	—	—	—					
Gaare et al. (1990)	Parallel‐arm CT (no information on financial support)	Volunteers selected among soldiers with CPITN ≤ 2 and large amount of calculus	88	20–25	NR	NR	Scaling with ultrasonic and hand instruments	NR	NR	Individual instruction on toothbrushing, 5‐min video demonstration for motivation	After PMPR	Full‐mouth except third molars	Patient	2 months	BOP score	—
			41	20–25	NR	NR	No PMPR	NR	NR	Individual instruction on toothbrushing, 5‐min video demonstration for motivation	After initial assessment					
Lim and Davies (1996)	Parallel‐arm RCT (supported by university)	Adults randomly sampled among employees of a telephone company	135	25–44	NR	NR	Scaling	Yes	NR	Personal instruction, self‐educational manual or video according to individual needs	NR	Full‐mouth	Patient	4 months	BOP score	Plaque score^b^
			176	25–44	NR	NR	No PMPR	—	—	Personal instruction, self‐educational manual or video according to individual needs	NR	Full‐mouth				
Somu et al. (2012)	Parallel‐arm RCT (no information on financial support)	Adults with chronic generalised gingivitis and least 24 natural teeth	10	20–45	NR	Non‐smokers	Scaling	NR	NR	No OHI (continuation of the routine oral hygiene method) + placebo gel to massage onto gums twice daily	—	Full‐mouth^a^	Patient	—	GI, PBI	Plaque score^b^
			10	20–45	NR	Non‐smokers	No PMPR	—	—	No OHI (continuation of the routine oral hygiene method) + placebo gel to massage onto gums twice daily	—	Full‐mouth^a^	Patient	—		

Abbreviations: BOP, bleeding on probing (as proposed by Ainamo and Bay 1975 and modified by Lang et al. 1986); CPITN, Community Periodontal Index of Treatment Needs (Ainamo et al. 1982): FQ, focused question; GI, gingival index (Löe and Silness 1963); GBS, gingival bleeding score (Cowell et al. 1975); NR, not reported; OHI, oral hygiene instruction; PBI, papillary bleeding index (Saxer and Mühlemann 1975); PMPR, professional mechanical plaque removal; RCT, randomised controlled trial.

^a^ Not explicitly reported in the article but inferred by the authors of the present review based on how the data are presented in the article.

^b^ Data based on the following scales: Quigley and Hein (1962) for Tan and Saxton (1978); O'Leary et al. (1972) for Lim and Davies (1996); Silness and Löe (1964) for Somu et al. (2012).

For the study by Albonni et al. (2021) contributing to FQ2, patient selection required age ≥ 16 years. However, the mean age and standard deviation of participants (Table 1a) indicated that the population could reasonably be considered adult; therefore, the study was included in the review. For this study, only data derived from patient‐level analyses (i.e., BOP score, treatment time, and PROMs) were extracted and included in the analyses.

The main PICOTS elements of the included studies are described narratively in the following section. The list of studies excluded after full text evaluation (along with reasons for exclusion) is reported in Appendix S2.

#### 3.1.1 Population

In one study (Mostafa et al. 2022), the population consisted of subjects with a diagnosis of plaque‐induced gingivitis performed according to the 2018 case definition (Trombelli et al. 2018; Chapple et al. 2018) or subjects without periodontitis presenting with gingival inflammation (Patil et al. 2015). In the other studies, it consisted of young adults with gingival inflammation without explicit information on the integrity of their periodontium (Tan and Saxton 1978; Gaare et al. 1990; Lim and Davies 1996; Somu et al. 2012; Narayanan et al. 2020; Shi et al. 2020), eventually including some mild periodontitis cases as stated in the patient selection criteria (Albonni et al. 2021; Mensi et al. 2022) or inferred indirectly by data on CAL (Assaf et al. 2007).

For the three studies reporting the average prevalence of BOP at baseline as derived from either full‐mouth (Gaare et al. 1990; Lim and Davies 1996) or half‐mouth assessments (Mensi et al. 2022), BOP prevalence was higher than the threshold (30%) currently used to define a generalised gingivitis case (Trombelli et al. 2018, Chapple et al. 2018). Among the other eight studies using parameters of gingival inflammation other than BOP, only one showed an average severity of baseline inflammation below the lowest score, indicating clinically detectable inflammatory changes in the gingival tissues (Tan and Saxton 1978).

The number of patients per treatment arm ranged between 8 (Mostafa et al. 2022) and 176 (Lim and Davies 1996).

#### 3.1.2 Intervention

All PMPR‐based interventions incorporated scaling, either generically reported as such (Lim and Davies 1996; Somu et al. 2012) or explicitly described as a scaling session performed with ultrasonic instruments (Assaf et al. 2007; Patil et al. 2015; Shi et al. 2020; Albonni et al. 2021; Mensi et al. 2022; Mostafa et al. 2022), hand instruments (Narayanan et al. 2020) or both (Tan and Saxton 1978; Gaare et al. 1990). For interventions in studies addressing FQ2, PMPR was implemented with air polishing with powder containing sodium bicarbonate (Patil et al. 2015), glycine (Shi et al. 2020) or erythritol–chlorhexidine (Albonni et al. 2021, Mensi et al. 2022), or application of a diode laser (Assaf et al. 2007). For studies addressing FQ3, professionally administered local adjuncts consisted of subgingival applications of an ornidazole–chlorhexidine gel (Narayanan et al. 2020) or gingival infiltrations of organic coconut oil or organic sesame oil with a microneedling technique (Mostafa et al. 2022).

PMPR was always administered in a single session. Information on local anaesthesia was reported only in one study addressing FQ3, where 2% lignocaine (1:80,000 adrenaline) infiltration was used in patients receiving one of the two Interventions, but not in those receiving the Comparator (Mostafa et al. 2022). Information on PMPR extension below the subgingival margin was explicitly reported in all studies addressing FQ2, all of which extended PMPR subgingivally (Assaf et al. 2007; Patil et al. 2015; Shi et al. 2020; Albonni et al. 2021; Mensi et al. 2022). When reported, the operator administering PMPR was a trained and certified operator (dentist or dental hygienist) (Tan and Saxton 1978; Shi et al. 2020; Mensi et al. 2022) or a trained undergraduate student (Albonni et al. 2021).

#### 3.1.3 Comparator

Comparators consisted of OHI alone (Tan and Saxton 1978; Gaare et al. 1990; Lim and Davies 1996), or no treatment, intended as the continuation of the routine oral hygiene procedures (Somu et al. 2012), for studies addressing FQ1; scaling alone (Assaf et al. 2007), scaling plus rubber cup polishing with a prophylaxis paste (Patil et al. 2015; Albonni et al. 2021; Mensi et al. 2022) or scaling plus glycine‐based air polishing followed by rubber cup polishing with a prophylaxis paste (Shi et al. 2020), for studies addressing FQ2; scaling without adjunctive local intra‐gingival infiltrations (Mostafa et al. 2022) or subgingival applications (Narayanan et al. 2020) of a gel for studies addressing FQ3.

#### 3.1.4 Outcomes

Primary outcomes used to assess gingival inflammation included the BOP score (according to the method of Ainamo and Bay 1975, as modified by Lang et al. 1986, who performed probing to the bottom of the sulcus/pocket; four studies), gingival index (GI) according to Löe and Silness (1963) (four studies), papilla bleeding index (PBI) (Mühlemann 1977; one study), sulcus bleeding index (SBI) according to Mühlemann and Son (1971) (two studies) or its modification (although proposed to evaluate the bleeding tendency of the marginal peri‐implant tissues) by Mombelli et al. (1987) (one study), gingival bleeding score (GBS) according to Cowell et al. (1975) (one study) and bleeding index (BI) (Mazza et al. 1981; one study).

In parallel‐arm RCTs, study outcomes were assessed at the full‐mouth level (Tan and Saxton 1978; Gaare et al. 1990; Lim and Davies 1996; Somu et al. 2012) except for one study which limited treatment application and outcome assessment to the six maxillary anterior teeth (Mostafa et al. 2022). All RCTs using a split‐mouth design applied a half‐mouth model (Assaf et al. 2007; Patil et al. 2015; Narayanan et al. 2020; Shi et al. 2020; Albonni et al. 2021; Mensi et al. 2022).

When available, secondary outcomes consisted of plaque scores (eight studies), probing depth (two studies), relative attachment levels measured between the bottom of the pocket and the edge of a resin stent (one study), stain index (one study), time for treatment administration (two studies) and patient perception and preference regarding the administered treatments (two studies).

#### 3.1.5 Timing

Among the included studies, five provided data on the primary outcome at observation intervals longer than 6 weeks, specifically at 2 months (Gaare et al. 1990), 3 months (Tan and Saxton 1978; Narayanan et al. 2020; Shi et al. 2020), 4 months (Lim and Davies 1996) and 6 months (Shi et al. 2020).

#### 3.1.6 Study Design

The present review included 10 RCTs (Tan and Saxton 1978; Lim and Davies 1996; Assaf et al. 2007; Somu et al. 2012; Patil et al. 2015; Mostafa et al. 2022; Narayanan et al. 2020; Shi et al. 2020; Albonni et al. 2021; Mensi et al. 2022) and 1 CT (Gaare et al. 1990). The included studies had a parallel‐arm (Tan and Saxton 1978; Gaare et al. 1990; Lim and Davies 1996; Somu et al. 2012; Mostafa et al. 2022) or split‐mouth (Assaf et al. 2007, Patil et al. 2015, Narayanan et al. 2020, Shi et al. 2020, Albonni et al. 2021; Mensi et al. 2022) design.

### 3.2 Summary of Main Findings

#### 3.2.1 What Is the Clinical Efficacy of PMPR in the Treatment of Gingivitis? (FQ1; Table 1b, Figure 2a,b, Appendix S3)

**TABLE 1b:** FQ1: Main results from RCTs and CTs evaluating the efficacy of PMPR alone with reference to no treatment (i.e., continuation of routine oral hygiene) or PMPR+OHI with reference to OHI alone.

Study	Treatment group	Primary outcomes (gingival inflammation scores)											Secondary outcomes			
		BOP score				GI		GBS			PBI		Plaque score^a^			
		Baseline	2–6 weeks	2 months	4 months	Baseline	2–6 weeks	Baseline	2–6 weeks	3 months	Baseline	2–6 weeks	Baseline	2–6 weeks	3 months	4 months
Tan and Saxton (1978)	OHI + PMPR							0.21	0.27^b^	0.30			2.34	1.83^b^	2.01^b^	
	OHI							0.24	0.29	0.30			2.17	2.09	2.25	
	PMPR							0.26	0.20^b^	0.30			2.30	2.03^b^	2.05^b^	
	No treatment							0.31	0.30	0.37^b^			2.16	2.13	1.94	
Gaare et al. (1990)	OHI + PMPR	63 (SE 1.4)	52^b^ (SE 1.8)	34^b^ (SE 1.5)	—											
	OHI	61 (SE 2.3)	43^b^ (SE 2.0)	36^b^ (SE 2.4)	—											
Lim and Davies (1996)	OHI + PMPR	35.4 (SD 21.1)	13.8^b^, ^c^ (SD 11.3)	—	16.0^b^, ^c^ (SD 11.5)								56.2 (SD 20.8)	32.6^b^, ^c^ (SD 16.1)		31.9^b^, ^c^ (SD 15.1)
	OHI	33.5 (SD 20.1)	22.0^b^, ^c^ (SD 16.7)	—	24.2^b^, ^c^ (SD 17.2)								52.1 (SD 21.4)	30.7^b^, ^c^ (SD 16.9)		31.7^b^, ^c^ (SD 15.9)
Somu et al. (2012)	PMPR					1.39 (SD 0.26)	1.09^b^ (SD 0.06)				1.26 (SD 0.34)	1.07^b^ (SD 0.22)	1.59 (SD 0.21)	1.14^b^ (SD 0.09)		
	No treatment					1.58 (SD 0.26)	1.48^b^ (SD 0.21)				1.26 (SD 0.37)	1.27 (SD 0.34)	1.55 (SD 0.25)	1.64 (SD 0.19)		

Abbreviations: BOP, bleeding on probing (as proposed by Ainamo and Bay 1975 and modified by Lang et al. 1986); FQ, focused question; GI, gingival index (Löe and Silness 1963); GBS, gingival bleeding score (Cowell et al. 1975); OHI, oral hygiene instruction; PBI, papillary bleeding index (Saxer and Mühlemann 1975); PMPR, professional mechanical plaque removal; RCT, randomised controlled trial; SD, standard deviation; SE, standard error (of the mean).

^a^ Data based on the following scales: Quigley and Hein (1962) for Tan and Saxton (1978); O'Leary et al. (1972) for Lim and Davies (1996); Silness and Löe (1964) for Somu et al. (2012).

^b^ Statistically significant intra‐group result (as compared to baseline).

^c^ Statistically significant inter‐group result (at the same timepoint).

**FIGURE 2:**
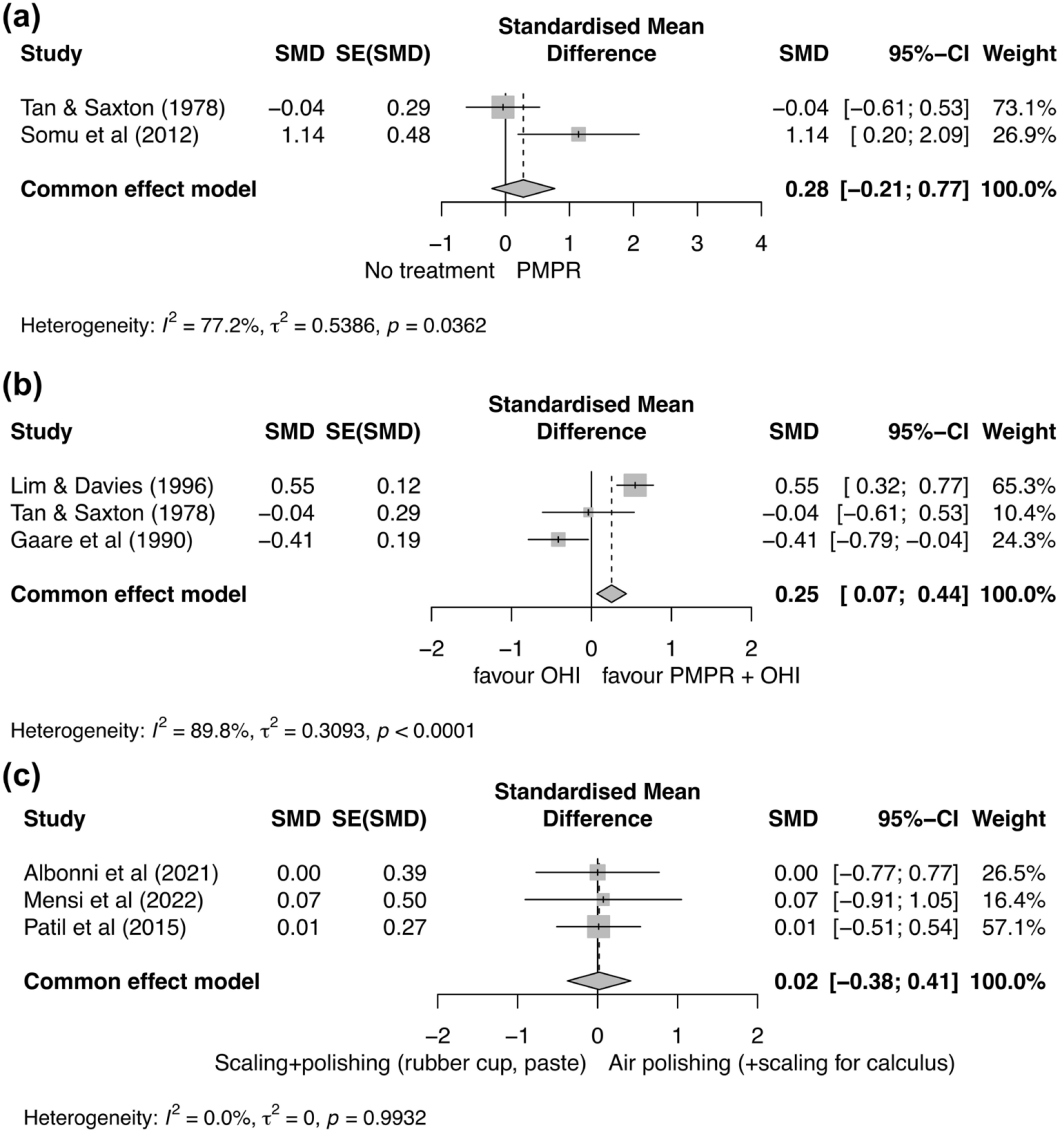
(a) FQ1: Effect size of PMPR versus no treatment on primary outcomes at 2–6 weeks as based on data extracted from the studies by Tan and Saxton (1978) and Somu et al. (2012). PBI was selected for the present meta‐analysis between the primary outcomes extracted from the study by Somu et al. (2012) (i.e., GI and PBI), since its definition is closer to gingivitis index. (b) FQ1: Effect size of PMPR + OHI versus OHI on primary outcomes (BOP score, GBS) at 2–6 weeks as based on data extracted from the studies by Tan and Saxton (1978), Gaare et al. (1990) and Lim and Davies (1996). (c) FQ2: Effect size of ultrasonic scaling + rubber cup polishing versus air polishing + ultrasonic scaling (the latter for calculus removal) on primary outcomes (BOP score, PBI, SBI, GI, mGI) at 2–6 weeks extracted from the studies by Patil et al. (2015), Albonni et al. (2021) and Mensi et al. (2022). In the study by Patil et al. (2015), the two treatment groups showed almost identical results for both primary outcomes, and SBI was used for the meta‐analysis.

**TABLE 2a:** FQ2: Characteristics of RCTs evaluating the efficacy of different interventions based on PMPR.

Study	Study design and financial support	Per protocol population (referred to the longest follow‐up visit reported in this table)					Treatment					Study outcomes and analysis		Follow‐up (in addition to the 2–6 week interval)	Primary outcomes (gingival inflammation scores)	Secondary outcomes
		Participants	*n*	Age (years)	Sex (F/M)	Smoking	PMPR modality in the intervention	PMPR modality in the comparator	PMPR extension below the gingival margin	Type of OHI	Timing of OHI	Sites for outcome assessment	Statistical unit			
Assaf et al. (2007)	Split‐mouth RCT (no information on financial support)	Adults with plaque‐induced generalised chronic gingivitis and least 20 natural teeth	22	21–50	14/8	Non‐smokers	Diode laser application followed by piezoelectric ultrasonic scaling	Piezoelectric ultrasonic scaling	Yes	NR	1 day before treatment	Half‐mouth	Patient	—	SBI	Plaque score^a^ PD RAL
Patil et al. (2015)	Split‐mouth RCT (no information on financial support)	Adults with all teeth (3rd molars excluded), PD ≤ 3 mm, and chronic marginal/papillary gingivitis	30	25.2 (SD 8.8)	16/14	NR	Ultrasonic scaling followed by air polishing using sodium bicarbonate powder	Ultrasonic scaling followed by‐polishing using a bristle brush, followed by rubber cup used with abrasive paste	Yes	None (patients continued their own routine oral hygiene procedures)	—	Half‐mouth	Patient	—	SBI GI	Plaque score^a^
Shi et al. (2020)	Split‐mouth RCT (supported by university)	Systemically healthy adults with gingivitis	33	27.6 (min 20 – max 38)	14/19	Non‐smokers	Ultrasonic scaling and air polishing with glycine powder	Ultrasonic scaling and air polishing with sodium bicarbonate and rubber cup polishing + paste	Yes	Correction of toothbrushing technique	Repeated every visit	3 teeth (central incisor, first premolar, first molar) per quadrant	Patient	3 and 6 months	BI	Plaque score^a^ PD Tooth stain
Albonni et al. (2021)	Split‐mouth RCT (no information on financial support)	Adults with gingivitis or Stage I periodontitis and least 20 natural teeth	13	24.62 (SD: 4.35; min 16–max 32)	5/8	Light smokers (< 5 cigarettes/day) eligible for the study	Air polishing with an erythritol‐chlorhexidine powder followed by piezoelectric ultrasonic scaling to remove calculus when required	Piezoelectric ultrasonic scaling and polishing with a rubber cup and fluoride‐containing paste	Yes	Modified Bass toothbrushing technique and dental floss	Immediately before treatment	Half‐mouth	Site^b^	—	BOP	Plaque score^a^ Treatment time PROMs
Mensi et al. (2022)	Split‐mouth RCT (supported by university)	Adults with BOP score > 25%, no sites with CAL > 3 mm and least 20 natural teeth	41	28.4 (SD 6.1; min 20–max 40)	20/21	30 non‐smokers, 11 smokers ≤ 10 cigarettes/day	Air polishing with an erythritol‐chlorhexidine powder followed by piezoelectric ultrasonic scaling to remove calculus when required	Piezoelectric ultrasonic scaling and polishing with a rubber cup and prophylaxis paste	Yes	Manual soft brush, interdental floss and regular sodium fluoride 0.24% w/w toothpaste	Immediately after treatment	Half‐mouth	Patient	—	BOP	Plaque score^a^ Treatment time PROMs

Abbreviations: BI, bleeding index (Mazza et al. 1981); BOP, bleeding on probing (as proposed by Ainamo and Bay 1975 and modified by Lang et al. 1986); F, females; FQ, focused question; GI, gingival index (Silness and Löe 1964); M, males; NR, not reported; OHI, oral hygiene instruction; PD, probing depth; PMPR, professional mechanical plaque removal; PROMs, patient‐reported outcome measures; RAL, relative attachment level; RCT, randomised controlled trial; SBI, sulcus bleeding index (Mühlemann and Son 1971); SD, standard deviation.

^a^ Data based on one of the following scales: Silness and Löe (1964) for Assaf et al. (2007) and Shi et al. (2020); Turesky et al. (1970) for Patil et al. (2015) and Albonni et al. (2021); O'Leary et al. (1972) for Mensi et al. (2022).

^b^ Although the study results were mainly based on a site‐level analysis, data on BOP score, treatment time and PROMs (which were reported at the patient level), as well as plaque score (which was statistically adjusted for Figure S5, as described in the Section 3.5), were retained for this review. On the other hand, data on papillary bleeding index (Mühlemann 1977), modified gingival index (Trombelli et al. 2004), calculus index and PD were not considered eligible for the review.

**TABLE 2b:** FQ2: Main results related to the primary outcome measures (gingival inflammation) of RCTs evaluating the efficacy of different interventions based on PMPR.

Study	PMPR modality	BOP score		BI				SBI		PBI		GI/mGI	
		Baseline	2–6 weeks	Baseline	2–6 weeks	3 months	6 months	Baseline	2–6 weeks	Baseline	2–6 weeks	Baseline	2–6 weeks
Assaf et al. (2007)	Diode laser + ultrasonic scaling							1.49 (SD 0.41)	0.33^a^ (SD 0.28)				
	Ultrasonic scaling							1.49 (SD 0.53)	0.38^a^ (SD 0.38)				
Patil et al. (2015)	Ultrasonic scaling and air polishing with sodium bicarbonate powder							1.41 (SD 0.83)	0.53^a^ (SD 0.46)			1.57 (SD 0.58)	0.81^a^ (SD 0.48)
	Ultrasonic scaling and polishing using a bristle brush, followed by rubber cup and abrasive paste							1.42 (SD 0.84)	0.55^a^ (SD 0.45)			1.58 (SD 0.59)	0.82^a^ (SD 0.46)
Shi et al. (2020)	Ultrasonic scaling and air polishing with glycine powder			3.35 (SD 0.7)	0.92^a^ (SD 0.21)	0.76^a^ (SD 0.16)	0.84^a^ (SD 0.17)						
	Ultrasonic scaling and air polishing with sodium bicarbonate and rubber cup polishing + paste			3.32 (SD 0.63)	0.80^a^ (SD 0.19)	0.74^a^ (SD 0.19)	0.80^a^ (SD 0.20)						
Albonni et al. (2021)	Air polishing with an erythritol‐chlorhexidine powder followed by piezoelectric ultrasonic scaling to remove calculus when required	0.40 (SD 0.490)	0.16^a^ (SD 0.363)							^c^	^c^	^c^	^c^
	Piezoelectric ultrasonic scaling and polishing with a rubber cup and fluoride‐containing paste	0.44 (SD 0.497)	0.20^a^ (SD 0.399)							^c^	^c^	^c^	^c^
Mensi et al. (2022)	Air polishing with an erythritol‐chlorhexidine powder followed by piezoelectric ultrasonic scaling to remove calculus when required	56.9% (CI: 51.1–63.5)	11.2%^b^ (CI: 7.7–16.5)										
	Piezoelectric ultrasonic scaling and polishing with a rubber cup and prophylaxis paste	56.7% (CI: 50.7–63.4)	14.8%^b^ (CI: 10.6–20.6)										

Abbreviations: BI, bleeding index (Mazza et al. 1981); BOP, bleeding on probing (as proposed by Ainamo and Bay 1975 and modified by Lang et al. 1986); CI, 95% confidence interval; FQ, focused question; GI, gingival index (Silness and Löe 1964); mGI, modified gingival index (Trombelli et al. 2004); PBI, papillary bleeding index (Mühlemann 1977); PMPR, professional mechanical plaque removal; SBI, sulcus bleeding index (Mühlemann and Son 1971); SD, standard deviation.

^a^ Statistically significant intra‐group result (as compared to baseline).

^b^ Statistically significant inter‐group result (at the same timepoint).

^c^ Data were available in the study but had been generated through a site‐level analysis and were therefore not considered for review or further analysis.

**TABLE 2c:** FQ2: Main results related to the secondary outcome measures of RCTs evaluating the efficacy of different interventions based on PMPR.

Study	PMPR modality	Plaque score^a^				Calculus index		PD (mm)				RAL (mm)		Stain index				Treatment time (min)	PROMs
		Baseline	2–6 weeks	3 months	6 months	Baseline	2–6 weeks	Baseline	2–6 weeks	3 months	6 months	Baseline	2–6 weeks	Baseline	2–6 weeks	3 months	6 months		
Assaf et al. (2007)	Diode laser + ultrasonic scaling	1.35 (SD: 0.44)	0.16^b^ (SD 0.11)					2.37 (SD 0.38)	1.99^b^ (SD 0.30)			6.14 (SD 1.11)	5.74^b^ (SD 1.00)						
	Ultrasonic scaling	1.36 (SD 0.42)	0.18^b^ (SD 0.10)					2.33 (SD 0.37)	2.04^b^ (SD 0.33)			6.01 (SD 1.04)	5.82^b^ (SD 1.00)						
Patil et al. (2015)	Ultrasonic scaling and air polishing with sodium bicarbonate powder	2.66 (SD 0.39)	1.79^b^ (SD 0.48)																
	Ultrasonic scaling and polishing using a bristle brush, followed by rubber cup plus abrasive paste	2.66 (SD 0.37)	1.80^b^ (SD 0.45)																
Shi et al. (2020)	Ultrasonic scaling and air polishing with glycine powder	1.67 (SD 0.56)	0.32 (SD 0.13)	0.31 (SD 0.14)	0.55 (SD 0.29)			2.29 (SD 0.48)	1.68^b^ (SD 0.41)	1.61^b^ (SD 0.45)	1.57^b^, ^c^ (SD 0.40)			0.58 (SD 0.56)	0.05^b^, ^c^ (SD 0.19)	0.07^b^, ^c^ (SD 0.21)	0.07^b^, ^c^ (SD 0.20)		
	Ultrasonic scaling and air polishing with sodium bicarbonate and rubber cup polishing + paste	1.69 (SD 0.55)	0.30 (SD 0.11)	0.30 (SD 0.12)	0.50 (SD 0.23)			2.34 (SD 0.50)	1.78^b^ (SD 0.37)	1.73^b^ (SD 0.37)	1.72^b^, ^c^ (SD 0.31)			0.58 (SD 0.55)	0.08^b^, ^c^ (SD 0.19)	0.11^b^, ^c^ (SD 0.23)	0.22^b^, ^c^ (SD 0.28)		
Albonni et al. (2021)	Air polishing with an erythritol‐chlorhexidine powder followed by piezoelectric ultrasonic scaling to remove calculus when required	2.33 (SD 1.153)^d^	2.36^b^ (SD 1.206)^d^			^e^	^e^	^e^	^e^									24.92^c^ (SD 9.260)	Preferred by the majority of patients due to less pain/discomfort, although this perception was not consistent with pain intensity that was assessed on a 100‐mm VAS
	Piezoelectric ultrasonic scaling and polishing with a rubber cup and fluoride‐containing paste	2.29 (SD 1.193)^d^	2.47^b^ (SD 1.221)^d^			^e^	^e^	^e^	^e^									34.08^c^ (SD 9.106)	
Mensi et al. (2022)	Air polishing with an erythritol‐chlorhexidine powder followed by piezoelectric ultrasonic scaling to remove calculus when required	65.0% (CI: 60.3–70.2)	12.7%^c^ (CI: 9.7–16.5)															18.39^b^ (CI: 17.42–19.38)	Preferred by the majority of patients due to higher perceived treatment quality and lower perceived discomfort during treatment
	Piezoelectric ultrasonic scaling and polishing with a rubber cup and prophylaxis paste	65.3% (CI: 59.7–71.4)	14.7%^c^ (CI: 11.1–19.5)															20.32^b^ (CI: 19.30–21.38)	

Abbreviations: CI, 95% confidence interval; FQ, focused question; PD, probing depth; PMPR, professional mechanical plaque removal; PROMs, patient‐reported outcome measures; RAL, relative attachment level, measured from the edge of the occlusal stent to the base of the pocket; SBI, sulcus bleeding index (Mühlemann and Son 1971); SD, standard deviation.

^**a**^ Data based on one of the following scales: Silness and Löe (1964) for Assaf et al. (2007) and Shi et al. (2020); Turesky et al. (1970) for Patil et al. (2015) and Albonni et al. (2021); O'Leary et al. (1972) for Mensi et al. (2022).

^b^ Statistically significant intra‐group result (as compared to baseline).

^c^ Statistically significant inter‐group result (at the same timepoint).

^d^ Data based on a site‐level analysis were available in the study and were statistically adjusted (see Section 3.5 for details) only to contribute to Figure S5.

^e^ Data were available in the study but had been generated through a site‐level analysis and were therefore not considered for review or further analysis.

**TABLE 3a:** FQ3: Characteristics of RCTs evaluating the efficacy of professionally administered adjuncts to PMPR.

Study	Study design and financial support	Population					Treatment					Study outcomes and analysis		Follow‐up (in addition to the 2–6 week interval)	Primary outcomes (gingival inflammation scores)	Secondary outcomes
		Participants	*n*	Age	Sex	Smoking	PMPR modality	PMPR extension below the gingival margin	Operator performing PMPR	Type of OHI	Timing of OHI	Sites for outcome assessment	Statistical unit			
Narayanan et al. (2020)	Split‐mouth RCT (no information on financial support)	Systemically healthy adults with moderate to severe gingivitis and no previous history of periodontal treatment	30	20–40	NR	Non‐smokers	Scaling and polishing with hand and ultrasonic instruments	Yes	NR	NR	Immediately following professional therapies	Half‐mouth	Patient	3 months	GI, mSBI	Plaque score^a^ Adverse events
							Scaling and polishing with hand and ultrasonic instruments followed by subgingival applications (baseline and day +7) of an ornidazole‐chlorhexidine gel	Yes	NR	NR	Immediately following professional therapies	Half‐mouth				
Mostafa et al. (2022)	Parallel‐arm RCT (supported by university)	Adults aged with moderate to severe plaque‐induced gingivitis (Chapple et al. 2018) involving the maxillary anterior sextant	8	29.71 (min 27–max 40)	4/4	Non‐smokers	Ultrasonic scaling	NR	NR	Proper tooth brushing techniques and flossing (no mouthwash prescribed)	Before professional therapies	Maxillary anterior sextant	Patient	—	GI	Plaque score^a^
			8	27.57 (min 22–max 36)	2/6	Non‐smokers	Ultrasonic scaling + gingival infiltrations of topical organic coconut oil	NR	NR	Proper tooth brushing techniques and flossing (no mouthwash prescribed) + not brush their teeth for 1 day after treatment + no acidic or hot beverages for 1 day	Professional therapies + additional instructions after the procedure	Maxillary anterior sextant				
			8	24.29 (min 16–max 34)	2/6	Non‐smokers	Ultrasonic scaling + gingival infiltrations of topical organic sesame oil	NR	NR	Proper tooth brushing techniques and flossing (no mouthwash prescribed) + not brush their teeth for 1 day after treatment+ no acidic or hot beverages for 1 day	Professional therapies + additional instructions after the procedure	Maxillary anterior sextant				

Abbreviations: FQ, focused question; GI, gingival index (Löe and Silness 1963); mSBI, modified sulcus bleeding index (Mombelli et al. 1987); NR, not reported; OHI, oral hygiene instruction; PMPR, professional mechanical plaque removal: RCT, randomised controlled trial; SD, standard deviation.

^a^ Data based on the scale proposed by Silness and Löe (1964).

**TABLE 3b:** FQ3: Main results from RCTs evaluating the efficacy of professionally administered adjuncts to PMPR.

Study	Treatment group	Primary outcomes (gingival inflammation scores)						Secondary outcomes			
		GI			mSBI			Plaque score^a^			Adverse events
		Baseline	2–6 weeks	3 months	Baseline	2–6 weeks	3 months	Baseline	2–6 weeks	3 months	Entire experimental phase
Narayanan et al. (2020)	PMPR + OHI	1.77 (SD 0.40)	0.80^c^ (SD 0.32)	0.78 (SD 0.23)	1.88 (SD 0.38)	1.02^c^ (SD 0.23)	0.96 (SD 0.23)	1.95 (SD 0.39)	0.98^c^ (SD 0.21)	0.95 (SD 0.27)	None
	PMPR + subgingival applications of an ornidazole‐chlorhexidine gel	1.87 (SD 0.26)	0.45^c^ (SD 0.21)	0.58 (SD 0.19)	1.80 (SD 0.351)	0.63^c^ (SD 0.17)	0.65 (SD 0.19)	1.87 (SD 0.36)	0.57^c^ (SD 0.14)	0.68 (SD 0.20)	None
Mostafa et al. (2022)	PMPR	1.67 (SD 0.33)	0.96^c^ (SD 0.76)					1.52 (SD 0.75)	1.34 (SD 1.23)		
	PMPR + gingival infiltrations of topical organic coconut oil	2.50 (SD 0.66)	1.06^b^, ^c^ (SD 1.08)					1.62 (SD 0.32)	0.48^b^ (SD 0.50)		
	PMPR + gingival infiltrations of topical organic sesame oil	2.01 (SD 0.55)	0.56^b^, ^c^ (SD 0.44)					1.64 (SD 0.80)	0.48 (SD 0.51)		

Abbreviations: FQ, focused question; GI, gingival index (Löe and Silness 1963); mSBI (Mombelli et al. 1987); OHI, oral hygiene instruction; PMPR, professional mechanical plaque removal: RCT, randomised controlled trial; SD, standard deviation.

^a^ Data based on the scale proposed by Silness and Löe (1964).

^b^ Statistically significant intra‐group result (as compared to baseline).

^c^ Statistically significant inter‐group result (at the same timepoint).

**TABLE 4:** Certainty assessment according to GRADE.

Focused Question 1	What is the clinical efficacy of PMPR in the treatment of gingivitis?									
Interventions	Sample size	Outcome	Included RCTs/CTs	Risk of bias	Inconsistency	Indirectness	Imprecision	Other considerations	SMD (95% CI)	Certainty
PMPR/No treatment	37/34	Gingivitis indices	Tan and Saxton (1978)	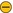	Serious^b^	Not serious	Not serious	None	0.28 (−0.21, 0.77)	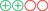 Low
			Somu et al. (2012)	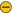 Serious^a^						
PMPR + OHI/OHI	248/239	Gingivitis Indices	Tan and Saxton (1978)	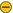	Serious^b^	Not Serious	Not serious	None	0.25 (0.07, 0.44)	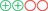 Low
			Lim and Davies (1996)	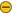						
			Gaare et al. (1990)	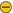 Serious^a^						

Focused Question 2	Among interventions based on PMPR, are some superior in terms of clinical outcomes, or do any allow for reduced treatment invasiveness while maintaining comparable clinical performance?									
Interventions	Sample size	Outcome	Included RCTs	Risk of bias	Inconsistency	Indirectness	Imprecision	Other considerations	SMD (95% CI)	Certainty
Scaling + rubber cup polishing/Scaling + air polishing	84/84	Gingivitis indices	Albonni et al. (2021)	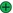	Not serious	Serious^d^	Not Serious	None	0.02 (−0.38, 0.41)	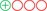 Very low
			Mensi et al. (2022)	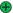						
			Patil et al. (2015)	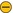 Very serious^c^						

*Note:* 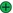 low risk of bias; 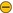 some concerns or moderate risk of bias.

Abbreviations: CI, confidence interval; CT, controlled trial; OHI, oral hygiene instruction; PMPR, professional mechanical plaque removal; RCT, randomised control trial; SMD, standardised mean difference.

^a^ Serious concern due to some concerns of risk of bias, potential limitations are likely to lower confidence in the estimate of effect.

^b^ Serious inconsistency across the studies detected from the minimally overlapping confidence intervals and significantly elevated *I* ^*2*^ statistic ≥ 75%.

^c^ Very serious due to study design biases (not fully reflected in ROB2), favouring air polishing, leading to lower confidence in the estimate of effect.

^d^ Serious due to the indirectness of the evidence, lowering confidence in the estimate of effect.

Two studies evaluated the efficacy of PMPR (consisting of professional scaling) versus no treatment (Tan and Saxton 1978; Somu et al. 2012) (Table 1b).

At 2–6 weeks, PMPR significantly decreased all gingival inflammation scores (i.e., GBS, GI and PBI) in patients continuing their routine oral hygiene procedures, and this effect was consistent with a significant decrease in the plaque score. Differently, for patients continuing their routine oral hygiene and not receiving PMPR, no significant changes in GBS were reported (Tan and Saxton 1978), and a significant decrease (although to a smaller magnitude compared to the PMPR group) was reported for GI, but not for PBI (Somu et al. 2012). No efficacy (expressed as ES) of PMPR versus no treatment was found on primary outcomes of gingivitis indices (GBS, PBI) at 2–6 weeks as derived from the two studies (Figure 2a), although a significant effect on plaque scores was observed (Appendix S3).

Only the study by Tan and Saxton (1978) included data on longer observation intervals (3 months), showing a relapse of gingival bleeding to baseline values for the PMPR group and to values significantly higher than baseline for the no‐treatment group. In patients receiving PMPR, the benefits of PMPR on supragingival plaque deposits were still evident at 3 months (Tan and Saxton 1978) (Table 1b).

Adjunctive PMPR led to greater improvements in the primary outcomes of gingival inflammation (BOP score, GBS) at 2–6 weeks in patients receiving OHI (Figure 2b). Similarly, a trend towards an adjunctive effect of PMPR on plaque scores was observed, although the confidence interval crossed the line of no effect by 0.01 (Appendix S3). Such findings were derived from two studies showing that PMPR + OHI significantly reduced BOP score to the same (Gaare et al. 1990) or a greater (Lim and Davies 1996) extent compared to OHI alone, and one study reporting no effect for PMPR + OHI at 2–6 weeks with respect to baseline values (Tan and Saxton 1978). In the latter study, the increase in gingival bleeding at 2–6 weeks was not explained by the plaque score, which showed a significant decrease. Differently, in the group receiving OHI alone, no significant changes in GBS and plaque scores were observed at 2–6 weeks from baseline (Tan and Saxton 1978) (Table 1b).

All studies reported data at intervals longer than 6 weeks. At 2 months following treatment administration, a further decrease in BOP was observed in one study, without inter‐group differences (Gaare et al. 1990). At 3 months, Tan and Saxton (1978) reported GBS values and plaque scores similar to the baseline values for both PMPR + OHI and OHI groups (Tan and Saxton 1978). In the study by Lim and Davies (1996), OHI significantly reduced BOP and plaque scores at 4 months with or without being combined with PMPR; however, the magnitude of the decrease in BOP scores was greater for the PMPR + OHI treatment group (Table 1b).

#### 3.2.2 Among the Interventions Based on PMPR, Are Some Superior in Terms of Clinical Outcomes, or Do Any Allow for Reduced Treatment Invasiveness While Maintaining Comparable Clinical Performance? (FQ2; Tables 2b and 2c, Figure 2c, Appendix S3)

In four studies, the PMPR modality used in the intervention group consisted of a combination of air polishing and ultrasonic scaling (US), the latter being used in all cases (Patil et al. 2015; Shi et al. 2020) or only for the removal of mineralised biofilm deposits (Albonni et al. 2021; Mensi et al. 2022). The comparator was US either alone (Patil et al. 2015; Albonni et al. 2021; Mensi et al. 2022) or in combination with air polishing with sodium bicarbonate (Shi et al. 2020), always followed by supragingival polishing with a rubber cup and a prophylaxis paste.

When combined with scaling, polishing with a prophylaxis paste and air polishing performed similarly for both primary outcomes of gingival inflammation (BOP score, PBI, SBI, GI, mGI) (Figure 2c) and plaque scores (Appendix S3) at 2–6 weeks. In the studies by Patil et al. (2015) and Albonni et al. (2021), a significant decrease in all parameters of gingival inflammation was observed at 2–6 weeks, with no significant difference between treatments (Table 2b), and plaque scores were similarly reduced in the former study (Table 2c). Differently, in the study by Mensi et al. (2022), a significantly lower BOP score (Table 2b) and plaque score (Table 2c) at 2–6 weeks were observed for the group receiving air polishing.

In the study by Shi et al. (2020), a similar significant decrease in all parameters of gingival inflammation and plaque score was observed at 2–6 weeks for patients receiving air polishing with glycine powder or air polishing with sodium bicarbonate and rubber cup polishing + paste following US (Table 2b).

In studies evaluating and reporting adverse events either during or following treatment administration (Patil et al. 2015; Albonni et al. 2021), one case of allergy was recorded some hours after treatment. Complete resolution was obtained within a few hours following the administration of an antihistamine tablet (Albonni et al. 2021).

When recorded, the average time needed for treatment administration was significantly shorter for air polishing + US compared to US + polishing with rubber cup and prophylaxis paste 24.92 vs. 34.08 min, respectively, for Albonni et al. (2021); 18.39 versus 20.32 min, respectively, for Mensi et al. (2022) (Table 2c).

PMPR performed by combining air polishing and US was preferred to US + polishing with rubber cup and prophylaxis paste by the majority (about two‐thirds) of patients (Albonni et al. 2021, Mensi et al. 2022). In one study, most patients self‐reported that this preference was due to less pain/discomfort, although this perception was not consistent with pain intensity that was assessed on a 100‐mm visual analogue scale (VAS) (Albonni et al. 2021). In another study, preference was mainly related to perceived treatment quality and perceived discomfort during treatment (Mensi et al. 2022) (Table 2c).

At 2–6 weeks, significantly greater reductions in tooth staining were observed for PMPR with US and air polishing with glycine powder in the study by Shi et al. (2020). Such a difference was observed even at 3 and 6 months, while the plaque score did not show significant inter‐group differences (Shi et al. 2020) (Table 2c).

Adjunctive efficacy of the diode laser was investigated in patients receiving US and OHI (Assaf et al. 2007). In quadrants assigned to the experimental treatment, laser (810 nm; repeated beam, 0.2 s on/0.3 s off, for an average of 15 s for each tooth; output power of 1.0 W) was applied approximately 30 min before US. Although treatment significantly improved all the investigated parameters (including SBI and plaque scores) at 2–6 weeks, no adjunctive clinical efficacy of diode laser application was observed (Assaf et al. 2007) (Tables 2b and 2c).

#### 3.2.3 Can the Professional Administration of Adjunctive Therapies Enhance the Clinical Outcomes of PMPR‐Based Interventions? (FQ3; Table 3b)

In a split‐mouth RCT, the adjunctive efficacy of two subgingival applications of an ornidazole–chlorhexidine containing gel was evaluated in patients receiving supragingival and subgingival scaling and polishing, and OHI (Narayanan et al. 2020). In areas assigned to experimental treatment, the ornidazole–chlorhexidine gel was applied subgingivally after PMPR, until it completely filled the sulcus, using a 23 gauge needle. Gel application was repeated after 7 days. Greater reductions in mSBI, GI and plaque scores were recorded at 2–6 weeks and 3 months when the gel was used as adjunct to PMPR; however, only inter‐group differences at 2–6 weeks reached statistical significance. Gel administration was not associated with adverse events (Narayanan et al. 2020).

A parallel‐arm RCT evaluated the adjunctive efficacy of topical gingival infiltrations (performed using a microneedling technique) of an organic coconut oil or an organic sesame oil in patients receiving US at the six maxillary anterior teeth in combination with OHI (Mostafa et al. 2022). At 2–6 weeks, the two experimental groups showed significant reductions in GI compared to baseline, while the control group (receiving only scaling and OHI) did not. This effect was explained by a significant reduction in plaque scores only in the group receiving topical coconut oil (Mostafa et al. 2022).

### 3.3 Reporting Standards and Risk of Bias

**FIGURE 3:**
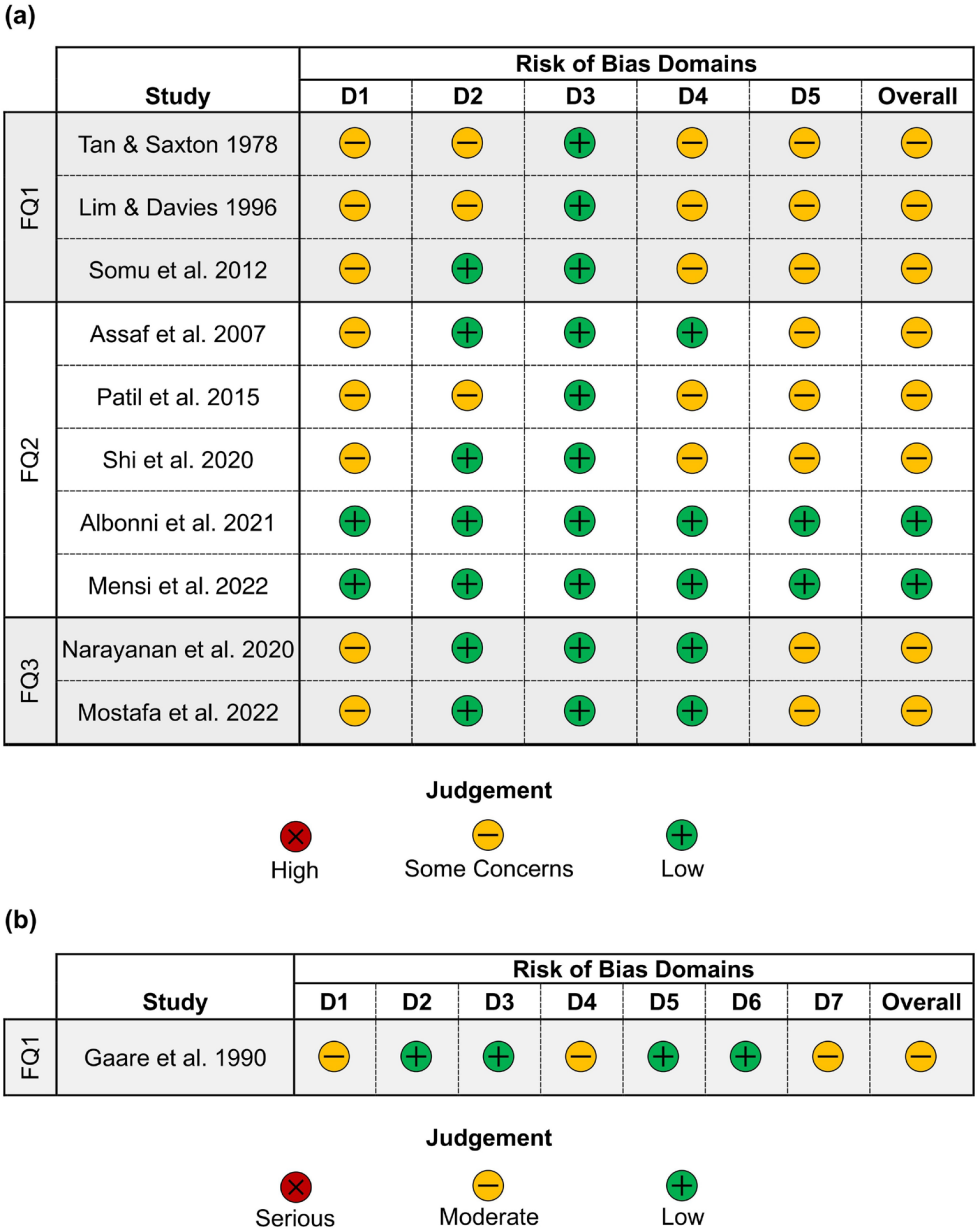
(a) Risk of bias (Sterne et al. 2019) for each included RCT (Cochrane risk of bias tool, ROB 2.0). (b) Risk of bias (Sterne et al. 2016) for the only included CT (Cochrane risk of bias tool, ROBINS‐I).

The risk of bias summary is provided in Figure 3a,b. Among the 10 included RCTs, 8 were classified as having ‘some concerns’ mainly due to bias arising from either the randomisation process (Domain 1) or the selection of the reported outcome(s) (Domain 5). Meanwhile, two studies were considered at low risk of bias (Albonni et al. 2021, Mensi et al. 2022).

The single CT (Gaare et al. 1990) that was included in the review was classified as ‘moderate’ for risk of bias.

### 3.4 Certainty Assessment and Strength of Recommendations

Table 4 summarises the certainty assessment using the GRADE framework, based on the meta‐analyses. While all but one of the included studies were RCTs, a majority were evaluated as having some concerns regarding the risk of bias (Figure 3), thereby lowering the certainty of evidence by at least one level. For FQ1, the certainty of evidence was further lowered by one level because of inconsistency across the studies. Thus, a low certainty of evidence supported the comparable efficacy of PMPR and no treatment (no significant difference according to meta‐analysis, Figure 2a). Low certainty evidence also supported the statistically significant benefit of PMPR + OHI in the management of gingivitis versus OHI alone according to meta‐analysis (Figure 2b). For FQ2, a very low certainty of evidence supported the comparable efficacy of PMPR performed using US plus rubber cup polishing versus air polishing plus US (with US for calculus removal) for managing gingivitis (Figure 2c). Because of the heterogeneous nature of adjuncts to PMPR that were evaluated (FQ3), the efficacy of each adjunct was assessed individually in separate studies, precluding a meta‐analysis and thus the GRADE assessment was not performed.

## 4 Discussion

To the best of our knowledge, this is the first systematic review to evaluate the efficacy of PMPR in treating naturally occurring, dental biofilm–induced gingivitis in adults without local plaque‐retentive factors (e.g., orthodontic appliances) and free from systemic diseases (e.g., Down's syndrome) or conditions (e.g., altered passive eruption) that may affect gingival or periodontal tissues and treatment response. Emphasis was placed on the indication for PMPR, the modality of its use and its implementation with local adjuncts. The global relevance of this topic is reflected in a health economics evaluation of different treatment options for periodontitis, which identified the most beneficial scenario as the elimination of incident gingivitis. Importantly, the modelling also showed that limiting gingivitis treatment coverage to only 10% of cases (a scenario in which fewer patients with gingivitis are treated, resulting in more progressing to periodontitis) would significantly increase costs and decrease healthy life years at both individual and societal levels (Economist Intelligence Unit 2021).

Within FQ1, the evidence comparing PMPR with no treatment consisted of two RCTs (Tan and Saxton 1978; Somu et al. 2012). Based on effect size, no significant efficacy of PMPR versus no treatment was observed for the primary outcome of gingival inflammation in patients maintaining ineffective (as indicated by plaque scores reported in Table 1b) routine oral hygiene procedures (Figure 2a). Data from one study (Tan and Saxton 1978) showed that, at 3 months, gingival bleeding relapsed to baseline levels in the PMPR group and to values significantly higher than baseline in the no‐treatment group. Despite this, a significant benefit of PMPR compared with no treatment was reported for plaque scores at 2–6 weeks (Appendix S3), and this benefit persisted at 3 months. Overall, the available evidence suggests that gingivitis treatment does not benefit from PMPR in patients continuing with an ineffective plaque control regimen; in such cases, however, PMPR may help prevent further exacerbation of the disease. Based on the detrimental effects of persistent gingival inflammation on periodontal tissues as documented histologically and clinically (Brecx et al. 1988; Lang et al. 2009), however, the use of PMPR alone does not seem to be a reasonable option from a practical perspective.

The second meta‐analysis performed within FQ1 favoured (although with low certainty of evidence) PMPR + OHI over OHI alone for the primary outcome of gingival inflammation (Figure 2b). This result was derived from three studies with a significant level of heterogeneity: one favouring PMPR + OHI; one favouring OHI alone; and one showing no difference between the two treatments. In addition, a trend (not reaching statistical significance, as the 95% CI crossed the line of no effect by 0.01) towards a greater effect of PMPR + OHI was observed for plaque scores at 2–6 weeks (Appendix S3). Differences in the baseline severity and extent of gingivitis may partly help guide clinical interpretation of the meta‐analysis. For example, the very mild baseline inflammation (0.21–0.24 on a GBS scale of 0–3, where 1 indicates BOP after 30s) reported in Tan and Saxton (1978)—a study that found no significant effect for either treatment—suggests that the baseline inflammatory condition was not severe enough for the effects of PMPR (and OHI) to become evident. By contrast, the clinical scenarios in the other two studies (Gaare et al. 1990; Lim and Davies 1996) were compatible with the current case definition of generalised gingivitis, although with markedly different average baseline BOP scores (33.5%–35.4% in Lim and Davies 1996 vs. 61%–63% in Gaare et al. 1990). Unfortunately, the other information reported in Gaare et al. (1990) and Lim and Davies (1996) does not clearly explain why these two studies provided contrasting indications in the meta‐analysis. Overall, the available data seem to indicate the benefit of adjunctive use of PMPR to OHI in generalised gingivitis cases.

In cases receiving PMPR in addition to OHI, the timing of PMPR administration (i.e., concomitant with or following OHI) may represent a clinically relevant issue. In the studies included in this review, all comparisons of PMPR + OHI versus OHI involved delivering PMPR and OHI within the same session, thus limiting their findings to that scenario. Interestingly, in a second crossover phase (not included in the present review) of the RCT by Lim and Davies (1996), patients who had initially received OHI alone or PMPR + OHI were re‐allocated at 10 months: those who had received OHI alone initially were given PMPR, and those who had received PMPR + OHI initially received OHI alone. At 16 months, all patients were reassessed. Although all treatment groups showed significant reductions in BOP compared with controls, no significant differences were observed between the groups, suggesting that the sequence of treatment delivery was not clinically relevant (Lim and Davies 1996).

Within FQ2, the meta‐analysis of three studies showed, with a very low level of certainty, that there is no specific preferred modality for delivering PMPR between US + rubber cup polishing and air polishing + US (the latter only for calculus removal) (Figure 2c). This finding was consistent for the primary outcomes of gingival inflammation and plaque scores. Given the absence of differences in the incidence of adverse events, along with the significantly shorter treatment time and greater patient preference reported for air polishing + US, the combination of air polishing and ultrasonics (the latter used for calculus removal) could be preferable to US + rubber cup polishing in some cases, as it may reduce treatment time and increase patient acceptance while maintaining comparable clinical performance. However, this consideration requires further substantiation in other aspects, such as cost effectiveness.

Interestingly, another study also found no significant differences in reductions in gingival inflammation and plaque scores between patients receiving air polishing with glycine powder or air polishing with sodium bicarbonate, compared to rubber cup polishing + paste following US. Nevertheless, it reported the benefits of air polishing in stain reduction (Shi et al. 2020), suggesting that combining air polishing with smaller particles (~20–45 μm glycine) and US may maintain clinical effectiveness while enhancing aesthetic outcomes through improved stain removal.

Evidence from a single RCT showed no significant clinical benefit from the adjunctive use of a diode laser in the treatment of gingivitis with US (Assaf et al. 2007). This is consistent with previous clinical practice guidelines in other disease contexts, which have recommended against using lasers as adjuncts to subgingival instrumentation in initial therapy for periodontitis or in supportive periodontal care (Sanz et al. 2020). These findings emphasise the importance of providing clinically indicated therapy (e.g., PMPR + OHI) without the need to consider the addition of a diode laser, which may entail substantial costs for both the clinician and the patient.

Two studies examined professionally administered local adjuncts in gingivitis management with PMPR (FQ3), reporting the benefits of ornidazole–chlorhexidine gel and organic coconut/sesame oil (Narayanan et al. 2020; Mostafa et al. 2022). Both were rated as having moderate risk of bias. Differences in product type and delivery protocols precluded a meta‐analysis. Moreover, baseline gingivitis severity and treatment effect were not reported using BOP scores, limiting comparability with current gingivitis case definitions and treatment endpoints. Patient perception and cost versus benefit were not evaluated; also, the requirement for anaesthesia with organic coconut/sesame oil may restrict its applicability for dental hygienists in some countries. Overall, the evidence for FQ3 is insufficient to support the routine use of professionally administered local adjuncts in PMPR‐based gingivitis treatment.

### 4.1 Limitations

Our review has several limitations. The limited evidence on the investigated topic necessitated a certain degree of flexibility in the study selection criteria. This included accepting studies on young adults with gingival inflammation without explicit reference to participants' baseline diagnoses, as well as studies involving populations that partially included mild‐to‐moderate periodontitis cases (stage I–II). The identified studies exhibited high heterogeneity in the reported research methods. A wide variety of indices and scoring systems were employed, which increased the complexity of the analysis and posed challenges in making meaningful comparisons across studies. Analysis and reporting also contributed to heterogeneity, with patient‐level data being derived from full‐mouth assessments in some studies and partial‐mouth assessments in others. This heterogeneity affected the assessment of reporting quality and risk of bias and, consequently, the certainty of the evidence. In addition, assumptions were required in some statistical analyses to enable meta‐analyses because of incomplete or unclear data reporting in certain studies.

## 5 Conclusions

Within the aforementioned limitations, the findings of this systematic review indicate the following:

– In patients with gingivitis who continue an ineffective self‐performed oral hygiene regimen, PMPR does not significantly reduce gingival inflammation but may help prevent further disease progression over a 3‐month period. Conversely, PMPR demonstrates an adjunctive benefit in patients adhering to oral hygiene instructions, with selected cases of generalised gingivitis potentially benefiting from the combined approach (i.e., PMPR + OHI) (FQ1);
– In gingivitis patients, the combination of air polishing and US (the latter used only for calculus removal) is as effective as US plus rubber‐cup polishing, while being faster and generally preferred by patients. Diode laser therapy, however, provides no additional benefit over US alone (FQ2);
– Although some professionally administered local adjuncts have shown positive outcomes in gingivitis patients receiving ultrasonic PMPR, their broader clinical application is limited due to unresolved clinical issues, scarce data on their effect size (as measured by BOP reduction) and uncertain cost effectiveness (FQ3).

### 5.1 Implications for Practice

In healthy adults without local factors influencing plaque accumulation or the clinical manifestation of gingival inflammation, management of naturally occurring dental biofilm–induced gingivitis is recommended according to the following indications:

– PMPR should be considered for its potential to enhance OHI outcomes, and its adjunctive use may be particularly indicated in generalised gingivitis cases. In general, PMPR should be considered in cases failing to reach a periodontally healthy condition at a re‐evaluation visit after OHI implementation. However, in the presence of mineralised biofilm deposits, PMPR and OHI can be administered within the same session;
– Air polishing can be combined with US within PMPR protocols to reduce treatment time, potentially improving patient preference and comfort;
– The use of diode lasers to supplement ultrasonic PMPR, as well as the application of local adjuncts (primarily administered as subgingival applications or gingival infiltrations), is not currently supported by available evidence.

### 5.2 Implications for Research

- RCTs are required to further investigate the requirements for OHI and PMPR in patients with gingivitis, classified according the 2018 Classification;
- Conversion from gingivitis to periodontal health (as assessed using the full‐mouth BOP score) should be considered as the primary outcome measure of treatment regimens aimed at reducing gingival inflammation;
- Study protocols should be designed to increase certainty and reduce risk of bias through correct randomisation processes, allocation concealment, appropriate statistical accounting for drop‐outs and protocol deviations, blinding of examiners and protocol registration prior to study commencement;
- Studies should ensure adequate sample size based on robust power calculations.

## Author Contributions

R.F., A.S., L.T. and P.M.P. conceptualised and designed the review. A.S., R.J.J.C. and P.M.P. were involved in the literature search and/or article selection. Y.‐K.T. performed data analysis. R.F., A.S., L.T. and P.M.P. drafted the manuscript, which was reviewed and finalised by all authors after discussion with the coordinators and participants of Working Group 1, 21st European Workshop on Periodontology.

## Funding

The authors have nothing to report.

## Conflicts of Interest

The authors declare no conflicts of interest.

## Supporting information


**Appendix S1:** Strategies for electronic literature search on Medline, Elsevier Scopus and Embase databases.

**Appendix S2:** Excluded studies and the reasons for exclusion.

**Appendix S3:** (a) FQ1: effect size of PMPR versus no treatment on plaque scores at 2–6 weeks as based on data extracted from the studies by Tan and Saxton (1978) and Somu et al. (2012). (b) FQ1: effect size of PMPR + OHI versus OHI on plaque scores at 2–6 weeks as based on data extracted from the studies by Tan and Saxton (1978) and Lim and Davies (1996). (c) FQ2: effect size of ultrasonic scaling + rubber cup polishing versus air polishing + ultrasonic (the latter for calculus removal) on plaque scores at 2–6 weeks extracted from the studies by Patil et al. (2015) and Mensi et al. (2022).

## Data Availability

The data that support the findings of this study are available from the corresponding author upon reasonable request.
