# Supplementary material for: Clinical Efficacy of Interventions Based on Professional Mechanical Plaque Removal in the Treatment of Dental Biofilm–Induced Gingivitis: A Systematic Review and Meta‐Analysis
Source: J Clin Periodontol. 2026 Jan 13;53(4):572–95. doi: 10.1111/jcpe.70083 (PMC12972606; doi:10.1111/jcpe.70083)
Supplement: Supplementary file 1 — Appendix S1: Strategies for electronic literature search on Medline, Elsevier Scopus and Embase databases. [file JCPE-53-572-s002.docx]

**Appendix S1. Strategies for electronic literature search on** Medline, Elsevier Scopus^©^ and Embase^©^ databases.

Finalised search terms (Dated 2^nd^ May 2025):

|  | **Terms** | **Records** |
| --- | --- | --- |
| **Pubmed** | (Gingivitis [All Fields] OR Periodontal Diseases [MESH] OR Gingivitis [MESH] OR ((gum [Title/Abstract] or gingiv* [Title/Abstract] ) AND (inflam* [Title/Abstract] or bleed* [Title/Abstract] OR diseas* [Title/Abstract]))) AND (Dental Scaling [MESH] OR Dental Prophylaxis [MESH] OR (Guided biofilm [Title/Abstract] AND (removal [Title/Abstract] OR therapy [Title/Abstract] )) OR ((dent* [Title/Abstract] OR teeth [Title/Abstract] OR tooth [Title/Abstract] ) AND (scal* [Title/Abstract] OR polish* [Title/Abstract] OR prophylax* [Title/Abstract] OR air polish* [Title/Abstract] OR airflow [Title/Abstract] OR plaque removal [Title/Abstract] OR plaque control [Title/Abstract] )) OR Treatment [All Fields] OR therapy [All Fields]) AND (clinicaltrial[Filter] OR randomizedcontrolledtrial[Filter]) | 8487 |
| **Embase** | (gingivitis OR 'periodontal disease'/exp OR 'gingivitis'/exp OR ((gum OR gingiv*) AND (inflam* OR bleed* OR diseas*))) AND ('dental scaling'/exp OR 'dental prophylaxis'/exp OR ('guided biofilm' AND (removal OR therapy)) OR ((dent* OR teeth OR tooth) AND (scal* OR polish* OR prophylax* OR 'air polish' OR airflow OR 'plaque removal' OR 'plaque control')) OR treatment OR therapy) | 1802 (with Embase only, RCT and Clinical Trial filter) |
| **Scopus**  **(article title, abstract)** | ( gingivitis OR "periodontal diseases" OR ( ( gum OR gingiv* ) AND ( inflam* OR bleed* OR diseas* ) ) ) AND ( "dental scaling" OR "dental prophylaxis" OR ( "guided biofilm" AND ( removal OR therapy ) ) OR ( ( dent* OR teeth OR tooth ) AND ( scal* OR polish* OR prophylax* OR "air polish" OR airflow OR "plaque removal" OR "plaque control" ) ) OR treatment OR therapy ) | 3589 (with dentistry, clinical trial, and Randomized Control Trial filters) |
