# Supplementary material for: Clinical Efficacy of Interventions Based on Professional Mechanical Plaque Removal in the Treatment of Dental Biofilm–Induced Gingivitis: A Systematic Review and Meta‐Analysis
Source: J Clin Periodontol. 2026 Jan 13;53(4):572–95. doi: 10.1111/jcpe.70083 (PMC12972606; doi:10.1111/jcpe.70083)
Supplement: Supplementary file 2 — Appendix S2: Excluded studies and the reasons for exclusion. [file JCPE-53-572-s003.docx]

**Appendix S2.** Excluded studies and the reasons for exclusion.

| No. | Reference | Reason for exclusion |
| --- | --- | --- |
| **Electronic Search** | | |
| 1 | Abellán R, Gómez C, Iglesias-Linares A, Palma JC. Impact of photodynamic therapy versus ultrasonic scaler on gingival health during treatment with orthodontic fixed appliances. Lasers Surg Med. 2019;51(3):256-267. doi:10.1002/lsm.23035 | Orthodontic patients |
| 2 | Akram Z, Shafqat SS, Aati S, Kujan O, Fawzy A. Clinical efficacy of probiotics in the treatment of gingivitis: A systematic review and meta-analysis. Aust Dent J. 2020;65(1):12-20. doi:10.1111/adj.12733 | Study design |
| 3 | Al Nazeh A, Alshahrani A, Almoammar S, Kamran MA, Togoo RA, Alshahrani I. Application of photodynamic therapy against periodontal bacteria in established gingivitis lesions in adolescent patients undergoing fixed orthodontic treatment. Photodiagnosis Photodyn Ther. 2020;31:101904. doi:10.1016/j.pdpdt.2020.101904 | Orthodontic patients |
| 4 | Alhamoudi N, Abduljabbar T, Vohra F, Javed F. Influence of mechanical debridement with adjunct probiotic therapy on clinical status and salivary cortisol levels in patients with periodontal inflammation. Eur Rev Med Pharmacol Sci. 2023;27(18):8360-8370. doi:10.26355/eurrev_202309_33758 | Home-based administration of adjuncts |
| 5 | Alqerban A. Efficacy of antimicrobial photodynamic and photobiomodulation therapy against Treponema denticola, fusobacterium nucleatum and human beta defensin-2 levels in patients with gingivitis undergoing fixed orthodontic treatment: A clinic-laboratory study. Photodiagnosis Photodyn Ther. 2020;29:101659. doi:10.1016/j.pdpdt.2020.101659 | Orthodontic patients |
| 6 | Alshehri D, Alqerban A, Samran A. Treatment efficacy of photoactivated disinfection versus Salvadora persica gel in experimental gingivitis. Photodiagnosis Photodyn Ther. 2020;29:101641. doi:10.1016/j.pdpdt.2019.101641 | Experimental Gingivitis |
| 7 | Alwan AH, Alghazali MW, Hussain AA. Evaluation of Gingegel Gel for the Treatment of Gingivitis: A randomized clinical trial. Journal of Emergency Medicine, Trauma and Acute Care. 2023;2023(3 - Second Mustansiriyah International Dental Conference (MIDC 2023)) | Home-based administration of adjuncts |
| 8 | Aly E, Hafez HS, Labib AH, et al. Effect of low level laser therapy on gingival inflammation in patients undergoing fixed orthodontic treatment: A randomized clinical trial. Open Access Maced J Med Sci. 2020;8:139-145. | Orthodontic patients |
| 9 | Amoian B, Moghadamnia AA, Barzi S, Sheykholeslami S, Rangiani A. Salvadora Persica extract chewing gum and gingival health: improvement of gingival and probe-bleeding index. Complement Ther Clin Pract. 2010;16(3):121-123. doi:10.1016/j.ctcp.2009.11.002 | Underage patients |
| 10 | Andrade DP, Carvalho ICS, Gadoi BH, Rosa LCL, Barreto LMRC, Pallos D. Subgingival Irrigation with a Solution of 20% Propolis Extract as an Adjunct to Non-Surgical Periodontal Treatment: A Preliminary Study. J Int Acad Periodontol. 2017;19(4):145-151. | Periodontitis Patients |
| 11 | Aziz-Gandour IA, Newman HN. The effects of a simplified oral hygiene regime plus supragingival irrigation with chlorhexidine or metronidazole on chronic inflammatory periodontal disease. J Clin Periodontol. 1986;13(3):228-236. doi:10.1111/j.1600-051x.1986.tb01465.x | Periodontitis Patients |
| 12 | Baeshen HA, Alshahrani A, Kamran MA, Alnazeh AA, Alhaizaey A, Alshahrani I. Effectiveness of antimicrobial photodynamic therapy in restoring clinical, microbial, proinflammatory cytokines and pain scores in adolescent patients having generalized gingivitis and undergoing fixed orthodontic treatment. Photodiagnosis Photodyn Ther. 2020;32:101998. doi:10.1016/j.pdpdt.2020.101998 | Orthodontic patients |
| 13 | Baygin O, Tuzuner T, Ozel MB, Bostanoglu O. Comparison of combined application treatment with one-visit varnish treatments in an orthodontic population. Med Oral Patol Oral Cir Bucal. 2013;18(2):e362-e370. Published 2013 Mar 1. doi:10.4317/medoral.18261 | Orthodontic patients |
| 14 | Bretz WA, Valente MI, Djahjah C, do Valle EV, Weyant RJ, Nör JE. Chlorhexidine varnishes prevent gingivitis in adolescents. ASDC J Dent Child. 2000;67(6):399-374. | Underage Patients |
| 15 | Caygur A, Albaba MR, Berberoglu A, Yilmaz HG. Efficacy of glycine powder air-polishing combined with scaling and root planing in the treatment of periodontitis and halitosis: A randomised clinical study. J Int Med Res. 2017;45(3):1168-1174. doi:10.1177/0300060517705540 | Periodontitis Patients |
| 16 | Chapple IL, Walmsley AD, Saxby MS, Moscrop H. Effect of subgingival irrigation with chlorhexidine during ultrasonic scaling. J Periodontol. 1992;63(10):812-816. doi:10.1902/jop.1992.63.10.812 | Periodontitis Patients |
| 17 | Checchi L, Forteleoni G, Pelliccioni GA, Loriga G. Plaque removal with variable instrumentation. J Clin Periodontol. 1997;24(10):715-717. doi:10.1111/j.1600-051x.1997.tb00187.x | Quantitative outcomes of interest not reported |
| 18 | Chen X, Zhao Y, Xue K, Leng M, Yin W. Microbiological and clinical effects of probiotic-related Zeger therapy on gingival health: a randomized controlled clinical trial. BMC Oral Health. 2024;24(1):1086. doi:10.1186/s12903-024-04846-x | Home-based administration of adjuncts |
| 19 | de Almeida Silva Levi YL, Ribeiro MC, Silva PHF, et al. Effects of oral administration of Bifidobacterium animalis subsp. lactis HN019 on the treatment of plaque-induced generalized gingivitis. Clinical Oral Investigations. 2023;27(1):387-398. | Home-based administration of adjuncts |
| 20 | Deepshika S, Saravanan R, Sathya Sree V, Kannan P, Muthu K. Effects of Acupressure as an Adjunct to Oral Prophylaxis in Treatment of Gingivitis: A Randomized Case-control Study. J Nat Rem. 2022;22(1):85-90. | Home-based administration of adjuncts |
| 21 | Dos Santos GM, Botti MR. Influĕncia da profilaxia e do grau de higiene oral das gengivites [Influence of prophylaxis and degree of oral hygiene in gingivitis]. Rev Gaucha Odontol. 1975;23(3):220-226. | No PMPR |
| 22 | Dragoo MR. A clinical evaluation of hand and ultrasonic instruments on subgingival debridement. 1. With unmodified and modified ultrasonic inserts. Int J Periodontics Restorative Dent. 1992;12(4):310-323. | Study design |
| 23 | Fida A, Qureshi SA, Mumtaz F. Assessment of Herbal Preparation (Irimedadi Taila) An Adjunctive in Treating Plaque Caused Gingivitis. Pak J Med Health Sci. 2018;12(1):567-569. | Home-based administration of adjuncts |
| 24 | Fischman S, Picozzi A, Cancro L, Pader M. Influence of a chlorhexidine and a zinc mouthrinse on gingivitis. J Periodontol. 1975 Dec;46(12):710–714. doi: 10.1902/jop.1975.46.12.710. | Unclear Periodontal status |
| 25 | Frentzen M, Ploenes K, Braun A. Clinical and microbiological effects of local chlorhexidine applications. Int Dent J. 2002;52(5):325-329. doi:10.1002/j.1875-595x.2002.tb00879.x | Quantitative outcomes of interest not reported |
| 26 | Friel I, & Seefeld G. [Comparative clinical studies on reducing plaque and gingivitis] (Vol. 30). Germany 1980. | No PMPR |
| 27 | Furlaneto F, Levi Y, Ribeiro M, et al. Effects of oral administration of Bifidobacterium animalis subsp. lactis HN019 on the treatment of generalized gingivitis. J Clin Periodontol. 2022;49:173-174. | Home-based administration of adjuncts |
| 28 | Furlaneto F, Levi YLAS, Sávio DSF, et al. Microbiological profile of patients with generalized gingivitis undergoing periodontal therapy and administration of Bifidobacterium animalis subsp. lactis HN019: A randomized clinical trial. PLoS One. 2024;19(11):e0310529. Published 2024 Nov 11. doi:10.1371/journal.pone.0310529 | Home-based administration of adjuncts |
| 29 | Gómez C, Abellán R, Palma JC. Efficacy of photodynamic therapy vs ultrasonic scaler for preventing gingival inflammation and white spot lesions during orthodontic treatment. Photodiagnosis Photodyn Ther. 2018;24:377-383. doi:10.1016/j.pdpdt.2018.11.001 | Orthodontic patients |
| 30 | Greenstein G; Research, Science and Therapy Committee of the American Academy of Periodontology. Position paper: The role of supra- and subgingival irrigation in the treatment of periodontal diseases. J Periodontol. 2005;76(11):2015-2027. doi:10.1902/jop.2005.76.11.2015 | Study design |
| 31 | Hasturk H, Steed D, Tosun E, et al. Use of amnion-derived cellular cytokine solution for the treatment of gingivitis: A 2-week safety, dose-ranging, proof-of-principle randomized trial. J Periodontol. 2021;92(9):1317-1328. doi:10.1002/JPER.20-0800 | No PMPR |
| 32 | Iniesta M, Herrera D, Montero E, et al. Probiotic effects of orally administered Lactobacillus reuteri-containing tablets on the subgingival and salivary microbiota in patients with gingivitis. A randomized clinical trial. J Clin Periodontol. 2012;39(8):736-744. doi:10.1111/j.1600-051X.2012.01914.x | Home-based administration of adjuncts |
| 33 | Jankish A, Varghese J, Shenoy VP, U V, Khan S, Kamath V. Comparative evaluation of antimicrobial and anti-gingivitis effect of Ocimum tenuiflorum Linn. gel with 0.2% chlorhexidine gel – Randomized controlled clinical trial. J Herbal Med. 2021;29. | Home-based administration of adjuncts |
| 34 | Johnson RH, Rozanis J, Schofield ID, Haq MS. The effect of spiramycin on plaque accumulation and gingivitis. Dent J. 1978;44(10):456-460. | Experimental Gingivitis |
| 35 | Jones C, Milsom K, Ratcliffe P, Wyllie A, MacFarlane TV, Tickle M. Single-visit scale and polish for gingival health: The results of a practice-based rct. Clin Trials. 2011;8(4):528. | Repeated PMPR |
| 36 | Kang MS, Lee DS, Lee SA, Kim MS, Nam SH. Effects of probiotic bacterium Weissella cibaria CMU on periodontal health and microbiota: a randomised, double-blind, placebo-controlled trial. BMC Oral Health. 2020;20(1):243. Published 2020 Sep 2. doi:10.1186/s12903-020-01231-2 | Home-based administration of adjuncts |
| 37 | Kaslick RS, Tuckman MA, Chasens AI. Effect of topical vancomycin on plaque and chronic gingival inflammation. J Periodontol. 1973;44(6):366-368. doi:10.1902/jop.1973.44.6.366 | Home-based administration of adjuncts |
| 38 | Khosravi Samani M, Mahmoodian H, Moghadamnia A, Poorsattar Bejeh Mir A, Chitsazan M. The effect of Frankincense in the treatment of moderate plaque-induced gingivitis: a double blinded randomized clinical trial. Daru. 2011;19(4):288-294. | Underage patients |
| 39 | Kirschneck C, Christl JJ, Reicheneder C, Proff P. Efficacy of fluoride varnish for preventing white spot lesions and gingivitis during orthodontic treatment with fixed appliances-a prospective randomized controlled trial. Clin Oral Investig. 2016;20(9):2371-2378. doi:10.1007/s00784-016-1730-6 | Orthodontic patients |
| 40 | Kocher T, Topoll H. [Experimental studies on the effect of supra- and sub-gingival scaling with hand or ultrasonic instruments on the reduction of inflammation of the marginal gingiva in various oral hygiene methods]. Dtsch Zahnarztl Z. 1985;40(7):771-774. | Periodontitis Patients |
| 41 | Komara I, Alfa Winata E, Susanto A, Hendiani I. Periodontal tray application of chlorine dioxide gel as an adjunct to scaling and root planing in the treatment of chronic periodontitis. Saudi Dent J. 2020;32(4):194-199. | Home-based administration of adjuncts |
| 42 | Maisuoka T, Mikami T, Umcrawa K, Kumap T, Koja Y. The Effect of the Oral Probiotic on the Periodontal Status in Healthy Adults, Randomized, Double-blind, Placebo-controlled Parallel-group Comparative Study. Jpn Pharmacol Ther. 2020;48(2):197-202. | No PMPR |
| 43 | Malik NKA, Alkadhi OH. Effectiveness of mechanical debridement with and without antimicrobial photodynamic therapy against oral yeasts in children with gingivitis undergoing fixed orthodontic therapy. Photodiagnosis Photodyn Ther. 2020;31:101768. doi:10.1016/j.pdpdt.2020.101768 | Orthodontic patients |
| 44 | Maynor GB, Wilder RS, Mitchell SC, Moriarty JD. Effectiveness of a calculus scaling gel. J Clin Periodontol. 1994;21(5):365-368. doi:10.1111/j.1600-051x.1994.tb00728.x | Unclear Periodontal status |
| 45 | Mensi M, Scotti E, Sordillo A, Agosti R, Calza S. Plaque disclosing agent as a guide for professional biofilm removal: A randomized controlled clinical trial. Int J Dent Hyg. 2020;18(3):285-294. doi:10.1111/idh.12442 | PMPR with plaque disclosing agent vs PMPR without plaque disclosing agent |
| 46 | Mensi M, Scotti E, Sordillo A, Dalè M, Calza S. Air-polishing followed by ultrasonic calculus removal for the treatment of gingivitis: A 12-month, split-mouth randomized controlled clinical trial. Int J Dent Hyg. 2024;22(4):949-958. doi:10.1111/idh.12812 | Repeated PMPR |
| 47 | Miller DL, Hodges KO. Polishing the surface. A comparison of rubber cup polishing and airpolishing. Probe. 1991;25(3):103-109. | Quantitative outcomes of interest not reported |
| 48 | Montero E, Iniesta M, Rodrigo M, et al. Clinical and microbiological effects of the adjunctive use of probiotics in the treatment of gingivitis: A randomized controlled clinical trial. J Clin Periodontol. 2017;44(7):708-716. doi:10.1111/jcpe.12752 | Home-based administration of adjuncts |
| 49 | Morrow D, Wood DP, Speechley M. Clinical effect of subgingival chlorhexidine irrigation on gingivitis in adolescent orthodontic patients. Am J Orthod Dentofacial Orthop. 1992;101(5):408-413. doi:10.1016/0889-5406(92)70113-O | Orthodontic patients |
| 50 | Odor AA, Bechir ES, Forna DA. Effect of Hydrogen Peroxide Photoactivated Decontamination Using 940 nm Diode Laser in Periodontal Treatment: A Pilot Study. Photobiomodul Photomed Laser Surg. 2020;38(10):614-624. doi:10.1089/photob.2019.4718 | Periodontitis Patients |
| 51 | Ogaard B, Larsson E, Glans R, Henriksson T, Birkhed D. Antimicrobial effect of a chlorhexidine-thymol varnish (Cervitec) in orthodontic patients. A prospective, randomized clinical trial. J Orofac Orthop. 1997;58(4):206-213. doi:10.1007/BF02679961 | Orthodontic patients |
| 52 | Panhwar M, Rajpar SP, Abrar E, Alqutub M, Abduljabbar T. Effectiveness of chlorhexidine and metronidazole gels in the management of gingivitis. A clinical trial. Pak J Med Sci. 2021;37(5):1425-1429. | Home-based administration of adjuncts |
| 53 | Panhwar M, Rajpar SP, Abrar E, Alqutub M, Abduljabbar T. Effectiveness of chlorhexidine and metronidazole gels in the management of gingivitis. A clinical trial. Pak J Med Sci. 2021;37(5):1425-1429. | Home-based administration of adjuncts |
| 54 | Paschos E, Limbach M, Teichmann M, et al. Orthodontic attachments and chlorhexidine-containing varnish effects on gingival health. Angle Orthod. 2008;78(5):908-916. doi:10.2319/090707-422.1 | Orthodontic patients |
| 55 | Patil S, Varma SA, Suragimath G, Abbayya K, Zope SA, Kale V. Evaluation of Irimedadi Taila as an adjunctive in treating plaque-induced gingivitis. J Ayurveda Integr Med. 2018;9(1):57-60. | Home-based administration of adjuncts |
| 56 | Penmetsa GS, Kalyani Kondepudi L. Comparative evaluation of multiflower honey, jamun honey and chlorhexidine gluconate gel (0.2%) on clinical levels of dental plaque: One week randomized controlled clinical trial. Int J Pharma Bio Sci. 2017;8(4):P55-P60. | No PMPR |
| 57 | Penmetsa GS, Kalyani Kondepudi L. Comparative evaluation of multiflower honey, jamun honey and chlorhexidine gluconate gel (0.2%) on clinical levels of dental plaque: One week randomized controlled clinical trial. Int J Pharma Bio Sci. 2017;8(4):P55-P60. | Home-based administration of adjuncts |
| 58 | Peric M, Marhl U, Gennai S, Marruganti C, Graziani F. Treatment of gingivitis is associated with reduction of systemic inflammation and improvement of oral health-related quality of life: A randomized clinical trial. J Clin Periodontol. 2022;49(9):899–910. | Repeated OHI |
| 59 | Pinheiro SL, Donegá JM, Seabra LM, et al. Capacity of photodynamic therapy for microbial reduction in periodontal pockets. Lasers Med Sci. 2010;25(1):87-91. doi:10.1007/s10103-009-0671-6 | Periodontitis Patients |
| 60 | Pradeep AR, Kumari M, Priyanka N, Naik SB. Efficacy of chlorhexidine, metronidazole and combination gel in the treatment of gingivitis--a randomized clinical trial. J Int Acad Periodontol. 2012;14(4):91-96. | Home-based administration of adjuncts |
| 61 | Qadri T, Tunér J, Gustafsson A. Significance of scaling and root planing with and without adjunctive use of a water-cooled pulsed Nd:YAG laser for the treatment of periodontal inflammation. Lasers Med Sci. 2015;30(2):797-800. doi:10.1007/s10103-013-1432-0 | Periodontitis Patients |
| 62 | Ribeiro LSFE, Araujo NS, Zilli Vieira CL, Dos Santos JN, Cury PR. Impact of serum vitamin D levels on periodontal healing outcomes: A preliminary cohort study. Int J Dent Hyg. 2023;21(2):291-297. doi:10.1111/idh.12619 | Periodontitis Patients |
| 63 | Rotundo R, Nieri M, Cairo F, et al. Lack of adjunctive benefit of Er:YAG laser in non-surgical periodontal treatment: a randomized split-mouth clinical trial. J Clin Periodontol. 2010;37(6):526-533. doi:10.1111/j.1600-051X.2010.01560.x | Periodontitis Patients |
| 64 | Sapna N, Vandana KL. Evaluation of hyaluronan gel (Gengigel(®) ) as a topical applicant in the treatment of gingivitis. J Investig Clin Dent. 2011;2(3):162-170. doi:10.1111/j.2041-1626.2011.00064.x | Home-based administration of adjuncts |
| 65 | Schulz R., Seefeld G. [Therapy of gingivitis. Investigations of the effectiveness of preventive care program in dental practice]. Vol 39. Germany1989. | Repeated PMPR |
| 66 | Stein S, Schauseil M, Hellak A, Korbmacher-Steiner H, Braun A. Influence of Photobiomodulation Therapy on Gingivitis Induced by Multi-Bracket Appliances: A Split-Mouth Randomized Controlled Trial. Photomed Laser Surg. 2018;36(8):399-405. doi:10.1089/pho.2017.4404 | Orthodontic patients |
| 67 | Valente MI, Seabra G, Chiesa C, et al. Effects of a chlorhexidine varnish on the gingival status of adolescents. J Can Dent Assoc. 1996;62(1):46-48. | Underage Patients |
| 68 | Verma R, Tewari S, Singhal SR, Sangwan A. Effect of ethinyl estradiol/norethisterone acetate with and without scaling on periodontal status and high-sensitivity C-reactive protein levels in women with polycystic ovary syndrome having gingivitis: a randomized controlled trial. Quintessence Int. 2024;55(10):792-802. doi:10.3290/j.qi.b5751228 | Systemically ill patients |
| 69 | Vogel RI, Fink RA, Frank O, Baker H. The effect of topical application of folic acid on gingival health. J Oral Med. 1978;33(1):. | Home-based administration of adjuncts |
| 70 | Weaks LM, Lescher NB, Barnes CM, Holroyd SV. Clinical evaluation of the Prophy-Jet as an instrument for routine removal of tooth stain and plaque. J Periodontol. 1984;55(8):486-488. doi:10.1902/jop.1984.55.8.486 | Quantitative outcomes of interest not reported |
| 71 | Zanatta FB, Pinto TM, Kantorski KZ, Rösing CK. Plaque, gingival bleeding and calculus formation after supragingival scaling with and without polishing: a randomised clinical trial. Oral Health Prev Dent. 2011;9(3):275-280. | Unclear Periodontal status |
| 72 | Zeza B, Farina R, Pilloni A, Mongardini C. Clinical outcomes of experimental gingivitis and peri-implant mucositis treatment with professionally administered plaque removal and photodynamic therapy. Int J Dent Hyg. 2018;16(2):e58-e64. doi:10.1111/idh.12302 | Experimental Gingivitis |
| **Handsearch** | | |
| 1 | Asokan S, Emmadi P, Chamundeswari R. Effect of oil pulling on plaque induced gingivitis: a randomized, controlled, triple-blind study. Indian J Dent Res. 2009 Jan-Mar;20(1):47-51. | Home-based administration of adjuncts |
| 2 | Chawla, T. N., Nanda, R. S. & Kapoor, K. K. (1975) Dental prophylaxis procedures in control of periodontal disease in Lucknow (rural) India. J Periodont 46, 498–503. | Periodontitis patients |
| 3 | Dahlen, G., Lindhe, J., Sato, K., Hanamura, H. & Okamoto, H. (1992) The effect of supragingival plaque control on the subgingival microbiota in subjects with periodontal disease J Clin Periodont 19, 802–809. | Periodontitis patients |
| 4 | Eakle W, Boyd RL, Robertson PB, et al. Penetration of periodontal pockets with irrigation by a newly designed tip. J Dent Res 1988;67:400 (Abstr. 2295). | Home-based administration of adjuncts |
| 5 | Hetland, L., Midtun, N. & Kristoffersen, T. (1981) Effect of oral hygiene instructions given by paraprofessional personnel. Community Dentistry and Oral Epidemiology 10, 8–14. | Periodontitis patients |
| 6 | Hugoson, A., Lundgren, D., Asklow, B. & Borgklint, G. (2003) The effect of different dental health programme on young adult individuals. A longitudinal evaluation of knowledge and behaviour including cost aspects. Swedish Dental Journal 27, 115–130. | Repeated PMPR |
| 7 | Kho, P. Smales, F. C. & Hardie, J. M. (1985) The effect of supragingival plaque control on the subgingival microflora. J Clin Periodont. 12, 676–686. | Periodontitis patients |
| 8 | Lang, N. P., Cumming, B. R. & Löe, H.: Toothbrushing frequency as it relates to plaque development and gingival health. J Periodontol. 1973; 44: 396–405. | No PMPR |
| 9 | Larner JR, Greenstein G. Effect of calculus and irrigation tip design depth of subgingival irrigation. Int J Periodontics Restorative Dent 1993;13:288-297. | Home-based administration of adjuncts |
| 10 | Lightner, L. M., O'Leary, J. T., Drake, R. B., Crump, P. P. & Allen, M. F. (1971) Preventive periodontic treatment procedures: results over 46 months. Journal of Periodontology 42, 555–561. | Periodontitis patients |
| 11 | Listgarten, M. A., Schifter, C. C. & Laster, L. (1985) Comparative longitudinal study of 2 methods of scheduling maintenance visits: 4-year data.[erratum appears in Journal of Clinical Periodontology, 1989 Jul; 16(6):391]. Journal of Clinical Periodontology 12, 225–238. | Periodontitis patients |
| 12 | Lövdal, A., Arno, A., Schei, O. & Waerhaug, J. (1961) Combined effect of subgingival scaling and controlled oral hygiene on the incidence of gingivitis. Acta Odontol. Scand. 19, 533–553. | Periodontitis patients |
| 13 | Mojon, P., Rentsch, A., Budtz-Jorgensen, E. & Baehni, P. (1998) Effects of an oral health program on selected clinical parameters and salivary bacteria in a long-term care facility. European Journal of Oral Science 106, 827–834. | Institutionalized elderly |
| 14 | Newman MG, Flemmig TF, Nachnani S, et al. Irrigation with 0.06% chlorhexidine in naturally occurring gingivitis. II. 6-month microbiological observations. J Periodontol 1990;61:427-433. | Home-based administration of adjuncts |
| 15 | Parsons LG, Thomas LG, Southard GL, et al. Effect of sanguinaria extract on established plaque and gingivitis when supragingivally delivered as a manual rinse under pressure in an oral irrigator. J Clin Periodontol 1987;14:381-385. | Home-based administration of adjuncts |
| 16 | Petersen PE. Evaluation of a dental preventive program for Danish chocolate workers. Community Dent Oral Epidemiol. 1989 Apr;17(2):53-9. | Repeated PMPR |
| 17 | Petersilka G, Faggion CM Jr, Stratmann U, Gerss J, Ehmke B, Haeberlein I, Flemmig TF. Effect of glycine powder air-polishing on the gingiva. J Clin Periodontol. 2008 Apr;35(4):324-32. | Periodontitis patients |
| 18 | Schlagenhauf U, Jakob L, Eigenthaler M, Segerer S, Jockel-Schneider Y, Rehn M. Regular consumption of Lactobacillus reuteri-containing lozenges reduces pregnancy gingivitis: an RCT. J Clin Periodontol 2016; 43: 948–954. | No PMPR |
| 19 | Söderholm, G. (1979) Effect of a dental care program on dental health conditions. A study of employees of a Swedish shipyard. University of Lund, Malmo , Sweden . | Periodontitis patients |
| 20 | Söderholm, G., Nobreus, N., Attstrom, R. & Egelberg, J. (1982) Teaching plaque control (I). A 5-visit versus a 2-visit program. J Clin Periodont. 9, 203–213. | Periodontitis patients |
| 21 | Sturzenberger, O. P., Bosma, M. L., Moore, D. J. & Grossman, E. (1988) Clinical benefits of chlorhexidine in sustaining gingival health following prophylaxis. Journal of Clinical Dentistry 1, 24–27. | Home-based administration of adjuncts |
| 22 | Suomi, J. D., Greene, J. C. Vermillion, J. R., Doyle, J., Chang, J. J. & Leatherwood, E. C. (1971) The effect of controlled oral hygiene procedures on the progression of periodontal disease in adults. Results after third and final year. J Periodont 42, 152–160. | Periodontitis patients |
| 23 | Suomi, J. D., Smith, L. W., Chang, J. J. & Barbano, J. P.: Study of the effect of different prophylaxis frequencies on the periodontium of young adult males. J. Periodontol. 1973; 44: 406–410. | Periodontitis patients |
| 24 | Wolff L, Bakdash MB, Pihlstrom BL, Bandt C, Aeppli DM. The effect of professional and home subgingival irrigation with antimicrobial agents on gingivitis and early periodontitis. J Dent Hyg 1989;63:222-225,241. | Home-based administration of adjuncts |
| 25 | Zenthofer, A., Dieke, R., Dieke, A., Wege, K. C., Rammelsberg, P. & Hassel, A. J. (2013) Improving oral hygiene in the long-term care of the elderly - a RCT. Community Dentistry and Oral Epidemiology 41, 261–268. | Unclear periodontal status |
