# Supplementary figures and images for: Clinical Efficacy of Interventions Based on Professional Mechanical Plaque Removal in the Treatment of Dental Biofilm–Induced Gingivitis: A Systematic Review and Meta‐Analysis
Source: J Clin Periodontol. 2026 Jan 13;53(4):572–95. doi: 10.1111/jcpe.70083 (PMC12972606; doi:10.1111/jcpe.70083)

(a)

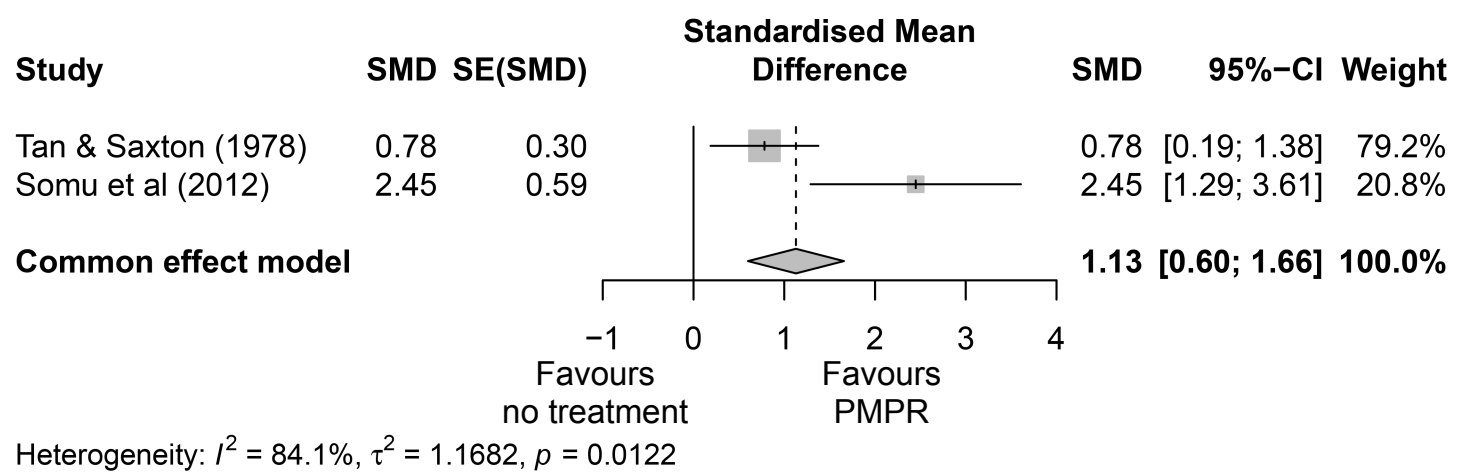

(b)

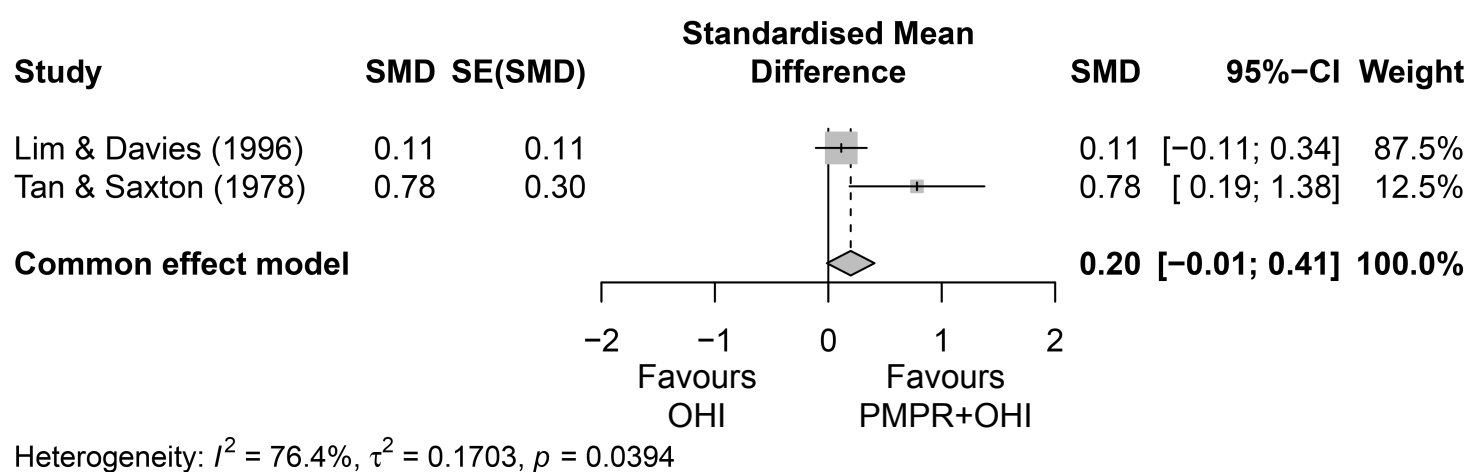

(c)

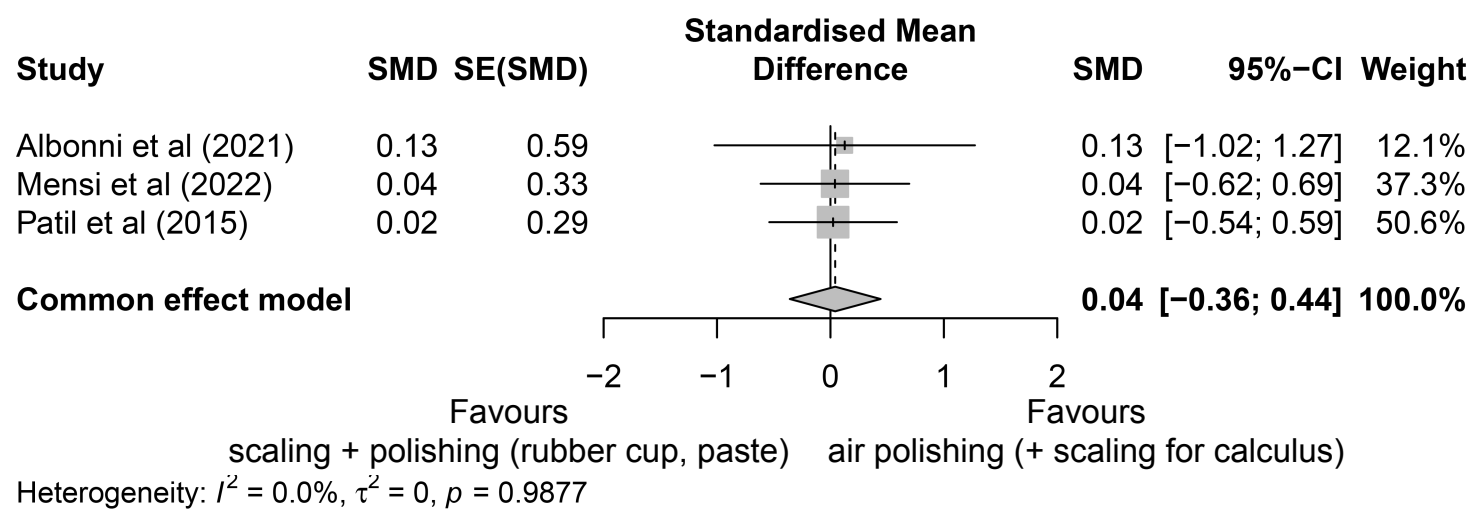

Supplement: Supplementary file 3 — Appendix S3: (a) FQ1: effect size of PMPR versus no treatment on plaque scores at 2–6 weeks as based on data extracted from the studies by Tan and Saxton (1978) and Somu et al. (2012). (b) FQ1: effect size of PMPR + OHI versus OHI on plaque scores at 2–6 weeks as based on data extracted from the studies by Tan and Saxton (1978) and Lim and Davies (1996). (c) FQ2: effect size of ultrasonic scaling + rubber cup polishing versus air polishing + ultrasonic (the latter for calculus removal) on plaque scores at 2–6 weeks extracted from the studies by Patil et al. (2015) and Mensi et al. (2022). [file JCPE-53-572-s001.pdf]
